# Computational reconstitution of spine calcium transients from individual proteins

**DOI:** 10.3389/fnsyn.2015.00017

**Published:** 2015-10-07

**Authors:** Thomas M. Bartol, Daniel X. Keller, Justin P. Kinney, Chandrajit L. Bajaj, Kristen M. Harris, Terrence J. Sejnowski, Mary B. Kennedy

**Affiliations:** ^1^Computational Neurobiology Laboratory, Howard Hughes Medical Institute, The Salk Institute for Biological StudiesLa Jolla, CA, USA; ^2^Center for Theoretical Biological Physics, University of CaliforniaSan Diego, La Jolla, CA, USA; ^3^Neurosciences Department, University of CaliforniaSan Diego, La Jolla, CA, USA; ^4^Department of Computer Science, Center for Computational Visualization, University of TexasAustin, TX, USA; ^5^Department of Neuroscience, Center for Learning and Memory, University of TexasAustin, TX, USA; ^6^Division of Biological Sciences, University of CaliforniaSan Diego, La Jolla, CA, USA; ^7^Division of Biology and Biological Engineering, California Institute of TechnologyPasadena, CA, USA

**Keywords:** dendritic spines, calcium pumps, calcium channels, synaptic calcium, synaptic plasticity

## Abstract

We have built a stochastic model in the program MCell that simulates Ca^2+^ transients in spines from the principal molecular components believed to control Ca^2+^ entry and exit. Proteins, with their kinetic models, are located within two segments of dendrites containing 88 intact spines, centered in a fully reconstructed 6 × 6 × 5 μm^3^ cube of hippocampal neuropil. Protein components include AMPA- and NMDA-type glutamate receptors, L- and R-type voltage-dependent Ca^2+^ channels, Na^+^/Ca^2+^ exchangers, plasma membrane Ca^2+^ ATPases, smooth endoplasmic reticulum Ca^2+^ ATPases, immobile Ca^2+^ buffers, and calbindin. Kinetic models for each protein were taken from published studies of the isolated proteins *in vitro*. For simulation of electrical stimuli, the time course of voltage changes in the dendritic spine was generated with the desired stimulus in the program NEURON. Voltage-dependent parameters were then continuously re-adjusted during simulations in MCell to reproduce the effects of the stimulus. Nine parameters of the model were optimized within realistic experimental limits by a process that compared results of simulations to published data. We find that simulations in the optimized model reproduce the timing and amplitude of Ca^2+^ transients measured experimentally in intact neurons. Thus, we demonstrate that the characteristics of individual isolated proteins determined *in vitro* can accurately reproduce the dynamics of experimentally measured Ca^2+^ transients in spines. The model will provide a test bed for exploring the roles of additional proteins that regulate Ca^2+^ influx into spines and for studying the behavior of protein targets in the spine that are regulated by Ca^2+^ influx.

## Introduction

One major goal of cellular neuroscience is to understand the molecular mechanisms that control the changes in synaptic strength governing learning and the formation of memory. It is well-established that the trigger for changes in synaptic strength in the CNS is influx of Ca^2+^ into dendritic spines (Franks and Sejnowski, 2002; Sjöström and Nelson, 2002). Thus, to understand the rules that govern changes in synaptic strength during normal brain function, and to clarify the interactions of pharmaceuticals with excitatory synapses, it is important to understand Ca^2+^ control mechanisms at a quantitative level. The basal level of Ca^2+^ in spines and dendrites and the size and timing of transient increases in Ca^2+^ during electrical activity are tightly controlled. Several decades of biochemical and biophysical work on individual enzymes and channels has resulted in detailed characterizations of the kinetic properties of proteins that are believed to regulate Ca^2+^ in spines and dendrites. At the same time, electrophysiologists have used fluorescent dyes that are sensitive to Ca^2+^ concentration to measure Ca^2+^ transients in spines produced by electrical signals in intact neurons. Here we use computer simulations to ask whether the aggregate behavior of the enzymes and channels believed to regulate Ca^2+^ in spines can account quantitatively for Ca^2+^ transients observed in spines in living neurons.

Synaptic strength is regulated by biochemical pathways driven by Ca^2+^ influx through N-methyl-D-aspartate-type glutamate receptors (NMDARs) and voltage-dependent Ca^2+^ channels (VDCCs) in dendritic spines (Kennedy, 2013). The direction of change in synaptic strength is critically dependent on the timing and amplitude of the Ca^2+^ influx (Magee and Johnston, 1997; Markram et al., 1997; Bi and Poo, 1998). Because NMDARs are sensitive to both voltage and glutamate, a few milliseconds difference in the timing of presynaptic release and postsynaptic depolarization can dramatically alter the amplitude and timing of Ca^2+^ transients and the direction of resulting changes in synaptic strength. It is particularly important to understand the complex mechanisms that regulate synaptic strength because mutations in several proteins that influence its regulation have been correlated with increased risk of mental diseases or with intellectual disability (Selkoe, 2002; Pinto et al., 2010; Hamdan et al., 2011; Sklar et al., 2011; Network and Pathway Analysis Subgroup of the Psychiatric Genomics, 2015).

As a first step, we have asked whether the properties of the crucial proteins that biochemists, pharmacologists, electrophysiologists and biophysicists have studied *in vitro*, together with our present understanding of their relative numbers and locations, are sufficient to explain the timing of Ca^2+^ transients observed experimentally *in situ* in stimulated spines. For a simpler system, biochemists would use a reconstitution experiment to try to reproduce the cellular behavior in question by combining purified proteins in a test tube (e.g., Kinoshita et al., 2001). However, full reconstitution experiments are not experimentally feasible for a complex function like control of Ca^2+^ concentration in spines because this function depends on the precise locations of the proteins on membranes and cytosolic structures.

As an alternative approach to experimental reconstitution, we generated a computer model in the program MCell (Kerr et al., 2008). The model incorporates realistic dendritic geometry, and includes the experimentally measured kinetics of the major Ca^2+^-handling proteins that are believed to control Ca^2+^ flux in spines of pyramidal neurons in area CA1 of the hippocampus (Table 1). It also incorporates their experimentally estimated numbers and spatial localizations. We show that application of a simulated electrophysiological stimulus to the model can produce Ca^2+^ transients that replicate the timing and amplitude of Ca^2+^ transients measured in spines in response to a back-propagating action potential (bAP; Sabatini et al., 2002). The successful reconstitution required adjustment of nine parameters within their range of experimental uncertainty.

**Table 1:** **Chemical kinetic rates and diffusion constants of molecules in model**.

**Parameter**	**Value**	**Temperature (°C)**	**Source**
Calcium			
Diffusion constant	2.2 × 10^−6^ cm^2^s^−1^	34	Allbritton et al., 1992
Glutamate			
Diffusion constant	2.2 × 10^−6^ cm^2^s^−1^	34	Bartol, 1992; Elowitz et al., 1999; Ellis, 2001
AMPAR		34	Jonas et al., 1993
k_C0C1_	9.18 × 10^6^ M^−1^s^−1^	34	
k_C1C0_	8520 s^−1^	34	
k_C1C2_	5.68 × 10^7^ M^−1^s^−1^	34	
k_C2C1_	6520 s^−1^	34	
k_C2O_	8480 s^−1^	34	
k_OC2_	1800 s^−1^	34	
k_C3C4_	2.54 × 10^6^ M^−1^s^−1^	34	
k_C4C3_	91.4 s^−1^	34	
k_C1C3_	5780 s^−1^	34	
k_C3C1_	78.4 s^−1^	34	
k_C2C4_	344 s^−1^	34	
k_C4C2_	1.45 s^−1^	34	
k_OC5_	35.4 s^−1^	34	
k_C5O_	8.0 s^−1^	34	
k_C4C5_	33.6 s^−1^	34	
k_C5C4_	380.8 s^−1^	34	
NMDAR: GluN2A/2B		34	Vargas-Caballero and Robinson, 2004
k_C0C1_	2 × 10^7^ M^−1^s^−1^	34	
k_C1C0_	11.0 s^−1^	34	
k_C1C2_	1.0 × 10^7^ M^−1^s^−1^	34	
k_C2C1_	22.0 s^−1^	34	
k_C2O_	93.0 s^−1^	34	
k_OC2_	183.2 s^−1^	34	
k_C2Ob_	97.0 s^−1^	34	
k_OC2b_	574 s^−1^	34	
k_C2D_	16.8 s^−1^	34	
k_DC2_	3.6 s^−1^	34	
k_*B*_(*V_m_*)	$1200e^{\left(\frac{-V_{m}}{17}\right)}$ s^−1^	34	Rate at 1 mM Mg^2+^
k_U_(*V_m_*)	$10800e^{\left(\frac{V_{m}}{47}\right)}$ s^−1^	34	
L-type and R-type VDCCs		34	Bischofberger et al., 2002
γ	3.72 pS	34	Church and Stanley, 1996 and Q_10_ 1.55
α*_i_*(*V_m_*)	$\alpha_{io}e^{\left(\frac{V_{m}}{V_{i}}\right)}$		
β*_i_*(*V_m_*)	$\beta_{io}e^{\left(\frac{V_{m}}{V_{i}}\right)}$		
α_1*o*_	8.08 ms^−1^	34	
α_2*o*_	13.4 ms^−1^	34	
α_3*o*_	8.78 ms^−1^	34	
α_4*o*_	34.7 ms^−1^	34	
β_1*o*_	5.76 ms^−1^	34	
β_2*o*_	12.6 ms^−1^	34	
β_3*o*_	16.3 ms^−1^	34	
β_4*o*_	3.68 ms^−1^	34	
*V*_1_	49.14 mV		
*V*_2_	42.08 mV		
*V*_3_	55.31 mV		
*V*_4_	26.55 mV		
k_Ca_(*V_m_*)	$\frac{\gamma V_{m}N_{A}\left(0.393-e^{\frac{-V_{m}}{80.36}}\right)}{2F\left(1-e^{\frac{V_{m}}{80.36}}\right)}$		
GluT: GLT1/GLAST		34	Otis and Jahr, 1998; Geiger et al., 1999
kT0T1	3.6 × 10^7^ M^−1^s^−1^	34	
kT1T0	360 s^−1^	34	
kT1T2	360 s^−1^	34	
kT2T0	51.4 s^−1^	34	
Immobile Calcium-Binding Protein (CBP)			Calibrated (see text)
Association rate	2.47 × 10^8^ M^−1^s^−1^	34	Calibrated (see text)
Dissociation rate	524 s^−1^	34	Calibrated (see text)
Diffusion constant	0 μm^2^s^−1^		
Calbindin		34	Nagerl et al., 2000
kM0M1	17.4 × 10^7^ M^−1^s^−1^	34	
kM1M2	8.7 × 10^7^ M^−1^s^−1^	34	
kM1M0	35.8 s^−1^	34	
kM2M1	71.6 s^−1^	34	
kH0H1	2.2 × 10^7^ M^−1^s^−1^	34	
kH1H2	1.1 × 10^7^ M^−1^ s^−1^	34	
kH1H0	2.6 s^−1^	34	
kH2H1	5.2 s^−1^	34	
Diffusion constant	0.28 × 10^−6^ cm^2^s^−1^	34	
PMCA		37	Penheiter et al., 2003; Brini and Carafoli, 2009
k_1_	1.5 × 10^8^ M^−1^s^−1^	37	
k_2_	15 s^−1^	37	
k_3_	12 s^−1^	37	
k_LEAK_	4.3 s^−1^		Gives 100 nM resting Calcium
NCX		34	Hilgemann et al., 1991
k_1_	3 × 10^8^ M^−1^s^−1^	34	
k_2_	300 s^−1^	34	
k_3_	600 s^−1^	34	
k_LEAK_	19.4 s^−1^		Gives 100 nM resting Calcium
SERCA2b		37	Higgins et al., 2006
k_X0X1_	2 × 10^8^ M^−1^s^−1^	37	
k_X1X0_	83.7 s^−1^	37	
k_X1X2_	1 × 10^8^ M^−1^s^−1^	37	
k_X2X1_	167.4 s^−1^	37	
k_X2Y2_	0.6 s^−1^	37	
k_Y2X2_	0.097 s^−1^	37	
k_X0Y0_	1.2 × 10^−3^	37	
k_Y0X0_	0.4 s^−1^	37	
k_Y0Y1_	60 × 10^−6^ M	37	Assume 60 μM [Ca^2+^] in ER
	×2 × 10^5^ M^−1^s^−1^		
k_Y1Y0_	30.02 s^−1^	37	
k_Y1Y2_	60 × 10^−6^ M	37	Assume 60 μM [Ca^2+^] in ER
	×1 × 10^5^ M^−1^s^−1^		
k_Y2Y1_	60.04 s^−1^	37	
Fluo4			
Association-rate	8.0 × 10^8^ M^−1^s^−1^	35	Naraghi, 1997
Dissociation-rate	240 s^−1^	35	*Kd* = 300 nM, Maravall et al., 2000
Diffusion constant	0.84 × 10^−6^ cm^2^s^−1^	34	Calculated (see text)
OGB1			
Association-rate	8.0 × 10^8^ M^−1^s^−1^	35	Naraghi, 1997
Dissociation-rate	160 s^−1^	35	*Kd* = 200 nM, Maravall et al., 2000
Diffusion constant	0.84 × 10^−6^ cm^2^s^−1^		Calculated (see text)

*The parameters used in the model of spine Ca^2+^ transients are listed, along with literature sources. Parameters were adjusted to reflect a range of 34–37°C, as described in the Methods and Results Sections. The usage of the parameters in kinetic models of each protein are shown in Figures 2, 3*.

The calibration of parameters was done by adding appropriate fluorescent Ca^2+^ indicators to the model, simulating the response of the indicators to a bAP, and then comparing the simulated responses to experimental results obtained in the presence of the same indicators (Sabatini et al., 2002; Scheuss et al., 2006). After calibration, we removed the indicators so that the model better approximated the *in vivo* state, and again applied simulated electrophysiological stimuli. The cytosolic Ca^2+^ transients produced in this way had a faster rise time and higher amplitude than those previously calculated from experimental fluorescence data (Maravall et al., 2000; Sabatini et al., 2002; Yasuda et al., 2003). We believe that our estimates are likely to be more accurate because the previous calculations were made with equations that were derived assuming steady-state conditions for Ca^2+^ binding to proteins in the spine (Neher and Augustine, 1992); but, our simulations show that a steady state is not reached during the rapid Ca^2+^ transients produced by a single bAP.

## Methods

The model was encoded in version 3.2 of the MCell Monte Carlo simulator (Kerr et al., 2008). MCell is a particle-based simulator of 3D reaction-diffusion systems. It permits the specification of a detailed cellular geometry and the assignment of discrete molecules, with their associated kinetics, to positions in the membrane, cytosol, or extracellular space. During the simulation, the diffusion of individual molecules is modeled as a pseudo-random walk constrained by the assigned diffusion constants. Fixed molecules have a diffusion constant of zero. Molecules can diffuse in two dimensions on the membrane, or in three dimensions in the cytosol and extracellular space. Interactions of molecules are detected by ray-tracing (Kerr et al., 2008), and binding events and/or first-order reactions are set to occur stochastically based on probabilities calculated from the kinetic rate constants assigned to each reaction. The global time step for all simulations was 1 μs. In MCell's reaction/diffusion algorithms, diffusion of individual molecules is simulated by a Monte Carlo random walk. The random walk is generated by scheduling each particle to move at a certain time in discrete spatial steps the length and direction of which are chosen by Monte Carlo sampling of the solution to the diffusion equation. During the simulation, time progresses by processing scheduled events in temporal order and the minimum elapsed time between events can be arbitrarily small. However, for internal accounting purposes the researcher specifies a global fixed timestep for keeping track of the states of most molecules. To balance computational efficiency and accuracy, molecules that move or react quickly can be scheduled to move/react with a finer temporal granularity than those that move/react more slowly. In our simulatins, we used this adaptive time stepping feature to simulate interaction of Ca^2+^ with a time step of 0.1 μs. Counting of reactions and molecules is described below. Data analysis was performed with custom Python scripts using Python-3.4, NumPy-1.9.2, SciPy-0.15.1, and matplotlib-1.4.3. Visualizations were created with the open source program Blender (blender.org) using the CellBlender addon. Both MCell and CellBlender are available at mcell.org.

### Geometry

**Figure 1:**
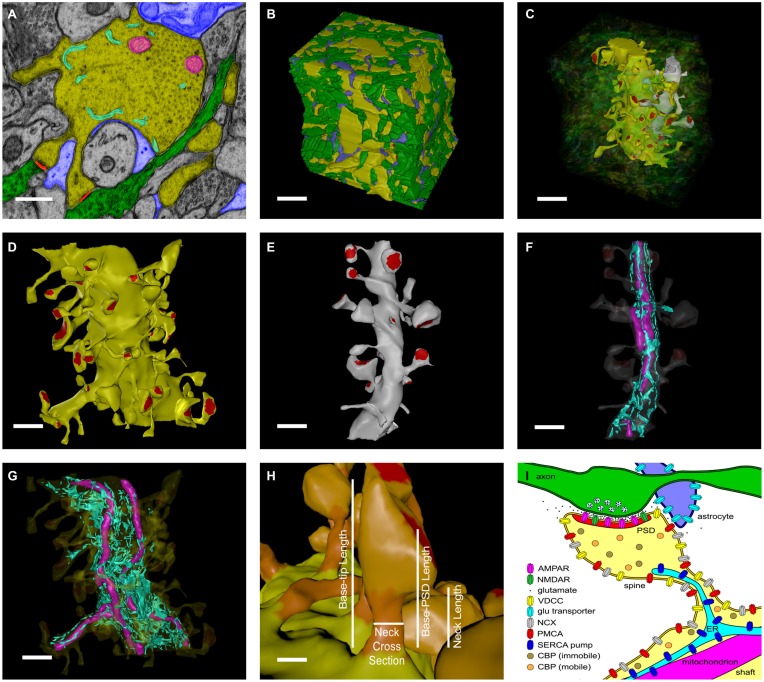
**Reconstruction of hippocampal neuropil**. **(A)** Hand segmentation of 2D-objects within a single EM thin section. The entire reconstruction consisted of 100 serial EM thin sections 50 nm thick. Each plasma membrane bound structure was traced in each thin section but only a few structures are highlighted here for illustrative purposes. A large apical dendrite is highlighted in yellow along with its enclosed endoplasmic reticulum (cyan) and mitochondria (magenta). Spines that are part of the principal dendrite, also shown in yellow, sometimes appear disjoint from the dendrite in a given thin section. Two axons that make synaptic contacts with two spines of the large apical dendrite in this plane of section are shown in green. PSDs and presynaptic active zones were annotated by enclosing them in a separate close-fitting contour (red). Processes of an astrocytic glial cell are shown in blue. Scale bar is 0.5 μm. **(B)** Reconstructed volume of neuropil. Dendrites are yellow. Axons are green. Astrocyte is blue. The extracellular space was adjusted so that the distance between components was 20 nm using an algorithm described in Kinney et al. (2013). Scale bar is 1 μm. **(C**) Two dendrites in the reconstructed volume were selected for simulations of glutamatergic synaptic transmission and postsynaptic calcium dynamics. A large apical dendrite is shown in yellow and a smaller branch dendrite is shown in light gray. The selected dendrites are shown in relation to one another embedded in the semitransparent neuropil. The synaptic contact areas (red) on the two selected dendrites were identified and labeled as shown in **(D,E**). Scale bar is 1 μm. **(D,E)** The PSD regions (red) of the large apical dendrite **(D)** and the smaller branch dendrite **(E)** were annotated. Scale bar is 1 μm. **(F,G)** Intracellular organelles of the small branch dendrite **(F)** and large apical dendrite **(G)**, including the reconstructed endoplasmic reticulum (cyan) and mitochondria (magenta). Scale bar is 1 μm. **(H)** The spine head (light-orange), neck (dark-orange), and dendritic shaft (yellow) were also annotated. Geometric measurements used in the analysis are shown as annotations. The neck cross section area was measured at a point halfway along the length of the neck. Scale bar is 0.25 μm. **(I)** Summary of all neuropil elements included in the model. The PSD was used to lasso a region of the spine surface for NMDAR and AMPAR placement. Presynaptic vesicles are shown for illustration only and were not included in simulations; instead glutamate was released directly into the synaptic cleft (see Methods Section). Note that about 19% of spines in the reconstruction contained ER protruding from the dendritic shaft into the spine neck and head.

We started with a 6 × 6 × 5 μm cube of neuropil from stratum radiatum of the hippocampus reconstructed from electron micrographs of 100 serial sections (Figure 1, see Mishchenko et al., 2010; Kinney et al., 2013). The process of reconstruction, adjustment of extracellular space, and creation of water-tight triangulated surfaces for computer simulation is described in detail in Kinney (2009), Kinney et al. (2013), and Edwards et al. (2014). The reconstruction of the extent and tortuosity of the extracellular space permits accurate modeling of the time-course of diffusion of glutamate through and out of the synaptic cleft. The cube is centered on two segments of dendrite that are entirely included within the volume. We used these two for the reconstitution study (Figure 1C). The larger proximal apical dendritic segment has a mean diameter of 2.1 μm and contains 72 spines that are located completely within the volume (Figure 1D). The smaller dendritic segment has a diameter of 0.8 μm and contains 16 complete spines (Figure 1E). In these two dendrites, endoplasmic reticulum (ER) and mitochondria were traced and included in the reconstruction and simulations (Figures 1F,G). Protrusions of the smooth endoplasmic reticulum (SER) into the spine neck and head are present in 14 spines in the larger dendrite and three in the smaller dendrite (about 19% of the spines of both dendrites). This percentage is comparable to that reported by Spacek and Harris (1997) and Cooney et al. (2002). Although all of the membranous structures in the cube were included in the simulations to allow for appropriate glutamate diffusion and re-uptake, we collected data about Ca^2+^ flux only from these two dendritic segments and their spines.

The locations of postsynaptic densities (PSDs) were defined by first tracing the contours of each PSD and active zone (AZ) on each original micrograph on which they appeared. The stack of such contours for a given PSD-AZ pair encloses the synaptic junction. The reconstructed contours include the intracellular extent of both the PSD and the AZ and intersect with the pre- and postsynaptic membrane associated with each. Therefore, we used the outer boundaries of the contours as a “3D-lasso” to tag surface mesh triangles representing the postsynaptic membrane and the location of the associated PSD (labeled in red for the large dendrite in Figure 1D).

MCell permits counting and tracking of molecules within a defined volume and also tracking of molecular fluxes across a defined surface. In the simulations reported here we tracked molecules of free Ca^2+^, Ca^2+^-bound to calcium binding proteins, and Ca^2+^ bound to indicator molecules in each spine head by creating “counting boxes” in which changing levels of each species were counted during the simulation. To count species in the spine head, a continuous form-fitting mesh was defined with its surface 5 nm from the outside surface of the spine head, and closed where the head connects with the neck. To count species in the entire spine, and to track the flux of Ca^2+^ through the base of the spine neck into the shaft, a second continuous form-fitting mesh was defined with its surface 10 nm from the outside surface of the spine (both head and neck), and closed at the place where the neck of the spine meets the dendritic shaft. Numbers of molecules within each counting box, and the flux between each spine and the shaft were measured at each time step of the simulations (1 μs), except for Ca^2+^, which was measured every 0.1 μs. All counts were recorded in an output file every 100 μs. Counts of molecules were sometimes converted to concentrations by converting to moles and dividing by the volume of the spine after subtracting the volume of any ER or mitochondria that intersected the sampling volume.

### Stimuli

The simulations reported here were initiated by release of glutamate from a single site on the presynaptic membrane (see Keller et al., 2008). The release was accompanied by a simulated voltage change in the postsynaptic membrane represented by 1 μs step changes in voltage read from a file generated in the program NEURON. Stimuli were simulated by adapting a NEURON model of a layer 5 neocortical pyramidal cell (Mainen et al., 1995). The NEURON model was implemented in pyramidal neuron “j4,” and included I_Na_, I_K_, I_KM_ (K channel blocked by Ach acting through the muscarinic receptor), I_KCa_ [Ca^2+^-dependent K channels (e.g., I_BK_, I_K_, I_SK_, and I_AHP_ currents), passive K (resting K channel), I_CaHVA_ (HVA-type Ca^2+^ channel), I_CaLVA_ (LVA, or T-type Ca^2+^ channel) in spines only], PMCA pump mediating Ca^2+^ decay. Its geometry consists of the soma, 11 primary dendrites, and 87 branches, divided into 164 compartments. Details are available in the NEURON ModelDB (https://senselab.med.yale.edu/ModelDB/ShowModel.cshtml?model=2488).

Backpropagating action potentials (bAP) were simulated by injecting current into the axon hillock and recording the voltages experienced at a spine located on a dendritic branch ~100 μm from the soma, as described in Keller et al. (2008). The NEURON model did not contain glutamate receptors. The excitatory postsynaptic potential (EPSP) was simulated by injecting current into the spine head as an alpha function such that a 5.8 mV peak EPSP was produced (see Keller et al., 2008). The peak of the EPSP occurred about 0.3 ms after release and decayed in ~2 ms. The EPSP plus bAP was simulated by injecting current into a spine to create an EPSP and 10 ms later injecting current into the axon hillock to initiate an action potential. The time-varying voltages recorded at the spine during these three stimuli were used to drive voltage-dependent transition rates in the models of VDCCs and NMDARs. The voltage changes alter the kinetic constants for relief of the Mg^2+^ block of the NMDARs, determine the flux of Ca^2+^ through open NMDAR and VDCC channels, and alter the kinetic constants that drive gating of VDCCs. An NMDAR channel opens and fluxes Ca^2+^ in the simulation only when glutamate is bound to the receptor at the time that the Mg^2+^ block is relieved.

### Proteins

Proteins were placed onto the membrane surfaces of the reconstructed dendrites or in the volume of the cytosol as shown in the cartoon in Figure 1I, and described in detail below. For each simulation initiated by a distinct random seed, proteins were placed randomly at a specified density or concentration, within each specified membrane or spatial compartment. Thus, the exact spatial locations of molecules and the sequences of stochastic binding events and enzymatic reactions vary randomly and depend on the seed used to initiate the simulation.

#### Sources of Ca^2+^ influx

##### Glutamate receptors

**Figure 2:**
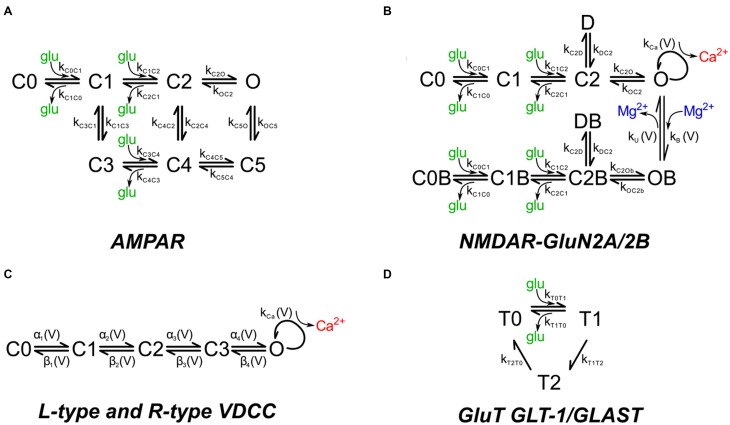
**Chemical kinetic schemes of receptors, Ca^2+^ channels, and glutamate transporter**. The kinetic rate constants for the reaction pathways are given in Table 1. **(A)** Glutamate-gated AMPAR with a mixture of GluA1 and GluA2 subunits and **(B)** glutamate-gated NMDA receptor with a mixture of GluN2A (NR2A) and GluN2B (NR2B) subunits were distributed on the dendritic spine head plasma membrane associated with the PSD. Mg^2+^ block was modeled by the asymmetric trapping block model of Vargas-Caballero and Robinson (2004). **(C)** Voltage-dependent Ca^2+^ channels on the spine head and neck are the sole source of calcium during backpropagating action potentials. **(D)** Glutamate transporter representing a combination of GLT-1 and GLAST transporter subtypes were distributed on the astrocytic glial processes.

AMPARs and NMDARs were placed into the postsynaptic membranes overlying the PSDs. The number of AMPARs is not a critical parameter in this study because the simulations are primarily concerned with Ca^2+^ influx through NMDARs and VDCCs. AMPARs are included in the simulation because AMPARs compete with NMDARs for binding of glutamate. Thus, the competition for binding of glutamate is modeled directly, allowing an accurate estimate of the activation of NMDARs. The density of AMPARs was set to 1200 μm^−2^ of PSD surface (120 receptors for a PSD of diameter 350 nm). *In vivo*, the number of AMPA receptors in spines is highly variable and is altered by synaptic plasticity (Nusser et al., 1998; Kharazia and Weinberg, 1999; Takumi et al., 1999; Racca et al., 2000). We have not included calcium-permeable AMPARs because they are relatively rare in excitatory synapses onto CA1 pyramidal neurons and are usually not a major source of Ca^2+^ flux. We used a kinetic model for activation of AMPARs by glutamate (Figure 2A; Table 1) based on an empirical model from Jonas et al. (1993) that describes recordings at 22–24°C from hippocampal slices of 15 to 24 day old rats. The model represents the behavior of AMPARs that contain a mixture of GluA1 and GluA2 subunits. We adjusted the parameters to 34°C using a Q_10_ of 2.0, because protein functions most commonly display a Q10 that is close to 2; thus, the adjustment is an approximation.

NMDARs were placed in the membranes overlying PSDs at a density of 150 μm^−2^ (15 receptors for a PSD of diameter 350 nm). This number is consistent with estimates of receptor numbers per PSD in the literature (e.g., 5–30 NMDARs per PSD, Spruston et al., 1995). We used the asymmetric trapping block kinetic model for activation of NMDARs, which includes distinct activation and deactivation rates for Mg^2+^ free and Mg^2+^ bound channels (Vargas-Caballero and Robinson, 2004; Figure 2B; Table 1). Their measurements were made at a temperature of 24–25°C; as we did for the AMPA receptors, we multiplied the rates for the NMDA receptors by a factor of 2 to adjust them to 34°C. The peak opening of NMDAR channels (whether blocked or unblocked) occurs at an average of 20 ms after glutamate release. The extra synaptic NMDARs on which this kinetic scheme is based contain GluN1 and a mixture of GluN2A and GluN2B subunits and are believed to have a higher proportion of GluN2B subunits than synaptic receptors (Stocca and Vicini, 1998).

We simulated the behavior of the NMDAR channel during a bAP, or during an EPSP followed by a bAP, by adapting a previously published model of the electrical behavior of a layer 5 neocortical pyramidal cell made in the program NEURON (Mainen et al., 1995), as described in detail in Keller et al. (2008), and above under *Stimulus*. Briefly, we used NEURON to generate a time series of dendritic membrane voltages for dendrites at the appropriate apical location, and a current–voltage (I–V) curve indicating the Ca^2+^ current through the open channel at each voltage. For the calculation, Ca^2+^ conductance was set at 4.5 pS (10% of 45 pS) and E_NMDA_ was set to 3 mV (Jahr and Stevens, 1993). The voltages were read by MCell and used to calculate probabilities of blocking and unblocking by Mg^2+^ from the equations for k_B_ and k_U_ in Table 1. When a receptor adopted an opened, unblocked state, the Ca^2+^ current through the pore was read from the I-V curve, and converted into the rate of Ca^2+^ flux per second according to the simple electrical equation,

(1) $$J=\frac{1}{2F}\times 6.02\times 10^{23}\frac{\text{ions}}{\text{mole}}$$

with flux, *J*, in ions/s, current, *I*, in amperes, and *F* as Faraday's constant. For example, if a Ca^2+^ current of 1 pA flows through a receptor, MCell would use a flux rate of 3.12 × 10^6^ s^−1^ to generate Ca^2+^ ions at the cytosolic mouth of the pore. To simulate the flux of individual ions through an ionotropic receptor, MCell randomly samples the Poisson distribution of time of the next flux event calculated from the flux rate constant. Thus, ions move one at a time through the open pore and the time between flux events has a Poisson distribution, generating the correct stochastic noise.

##### Voltage-dependent calcium channels (VDCCs)

Rapid, transient increases in dendritic Ca^2+^ occur when VDCCs in the membranes of spines and shafts are activated by excitatory postsynaptic potentials (EPSPs) or by bAPs. For the stimuli used in the simulations reported here, they are the largest source of Ca^2+^ influx. The precise complement of VDCCs in spines and shafts of pyramidal neurons is uncertain for two reasons. First, subtypes of VDCCs are sometimes difficult to separate pharmacologically. For example, CaV_1.3_ channels (one of two L-type channels) are difficult to distinguish from CaV_2.3_ channels (R-type) because both are resistant to inhibition by dihydropyridines (Higley and Sabatini, 2008). Second, neurons in different parts of the brain, such as the hippocampus and cortex, have distinct complements of Ca^2+^ channels in their dendrites (e.g., Markram et al., 1995; Koester and Sakmann, 2000; Bloodgood and Sabatini, 2007; Higley and Sabatini, 2008). Nonetheless, it is generally agreed that spines on pyramidal neurons in area CA1 of the hippocampus contain primarily R-type VDCCs with a small contribution from T-type channels; while the shafts of these same dendrites contain L, R, and T-type channels (Bloodgood and Sabatini, 2007).

The voltage sensitivity and activation kinetics of L and R-type channels are similar. However, R-type channels undergo slow inactivation during a depolarization of several 100 ms; whereas L-type channels do not (Magee and Johnston, 1995a). In this study, the activating stimuli were a single EPSP, and/or a single bAP. Therefore, we assumed that inactivation of the R-type VDCCs was not significant in the simulations reported here. This assumption permitted us to use a single kinetic scheme to represent both L- and R-type channels (Figure 2C). We adopted a five state kinetic scheme from Bischofberger et al. (2002; Figure 2C; Table 1). The scheme generated correct voltage sensitivity and activation curves at 23°C for L or R-type channels activated by single stimuli. We adjusted the parameters to 34°C using a Q_10_ of 2.0. A single channel conductance of 2.5 pS was reported by Church and Stanley (1996) based on measurements at 25°C of single channel conductances of VDCCs in chick ciliary ganglia at physiological Ca^2+^ concentration. As they suggested, we adjusted the conductance to 3.72 pS for 34°C using a Q_10_ of 1.55.

The probabilities of changes in state of individual channels and the Ca^2+^ ion influx through the open state was simulated by MCell in a manner similar to that described above for NMDA receptors. The time series of dendritic membrane voltages generated in NEURON was read by MCell and used to calculate α_*i*_ and β_*i*_ according to the equations in Table 1. The rate of Ca^2+^ flux through open channels at each membrane voltage (*k*_*Ca*_) was calculated from Equation (2) (Bischofberger et al., 2002).

(2) $$k_{Ca}(V_{m})=\frac{3.72V_{m}N_{A}\left[0.3993-e^{\left(\frac{-V_{m}}{80.36}\right)}\right]}{2F\left(1-e^{\left[\frac{V_{m}}{80.36}\right]}\right)}$$

The rate constant *k*_*Ca*_(*V*_*m*_) has the units s^−1^.

VDCCs have been estimated to have a density of ~2 μm^−2^ in apical dendrites when measured in membrane patches (Magee and Johnston, 1995a). We arrived at a calibrated density for VDCC's on spines of 1.20 μm^−2^ and on shafts of 2.67 μm^−2^, as described under Results Section.

T-type channels are also present in spines; however, they are largely inactivated at the resting potential that we used in our model, and that was used in the experiments of Sabatini et al. (2002; −65 to −70 mV; Fraser and MacVicar, 1991). Thus, they would contribute only a small proportion of Ca^2+^ influx after a single bAP stimulus (Magee and Johnston, 1995b). Therefore, they are not included in this model.

##### Glutamate transporters

After its release, the excitatory transmitter glutamate is removed from the extracellular space by glutamate transporters in the astrocyte membrane. We placed transporters in the astrocytes at a density of 10,000 μm^−2^ in agreement with the measurements of Lehre and Danbolt (1998). Crystal structures of related transporters suggest that its diameter is ~5 to 8 nm, indicating a range of maximum packing densities of 16,000–40,000 μm^−2^ (Yernool et al., 2004; Penmatsa et al., 2013). Thus, the glutamate transporter occupies a large fraction of the surface of astrocytes.

We used a simplified kinetic scheme (Figure 2D), implemented by Geiger et al. (1999), that reproduces the major binding, unbinding, and translocation characteristics of glutamate transport in cerebellar Purkinje cells, which is primarily attributed to EAAT4 transporters (Otis and Jahr, 1998). In hippocampal astrocytes, glutamate transport is mediated predominantly by EAAT1 (GLT-1) with a lesser contribution by EAAT2 (GLAST; Danbolt, 2001); but see Hu et al. (2003) on EAAT4 expression. Full kinetic models, with rate constants, for EAAT1, including Na^+^, K^+^, H^+^, and voltage dependence (Bergles et al., 2002), and for EAAT2 (Wadiche and Kavanaugh, 1998) have been published. However, the reduced model accurately reflects the slow turnover rate and the binding affinity for glutamate in the μM range of EAAT1 and EAAT2 (Arriza et al., 1994; Wadiche et al., 1995; Bergles et al., 2002). Thus, we believe it is sufficient for the present study in which we release single quanta of glutamate at individual synapses.

This model is primarily concerned with understanding the major proteins contributing to Ca^2+^ flux in the postsynaptic spine, therefore, we didn't include glutamate transporters on presynaptic axonal processes, although they have been shown to be present there in neuronal cultures and in perfusion fixed brain tissue (Wang et al., 1998; Chen et al., 2004). We also didn't include them on the postsynaptic membrane, although some studies have suggested they can modulate NMDA receptor activation (Diamond, 2001). In our reconstructed neuropil, not all spines are immediately adjacent to an astroglial process, therefore, the time-course of persistence of glutamate in the synaptic cleft is likely to be highly variable from spine to spine in the simulations (Ventura and Harris, 1999; Witcher et al., 2007). Glutamate transporters in the synaptic membrane would reduce this variability, but are not likely to have a large effect on the overall activation of NMDA receptors.

#### Cytosolic Ca^2+^ binding proteins

##### Immobile cytosolic Ca^2+^-binding proteins

**Figure 3:**
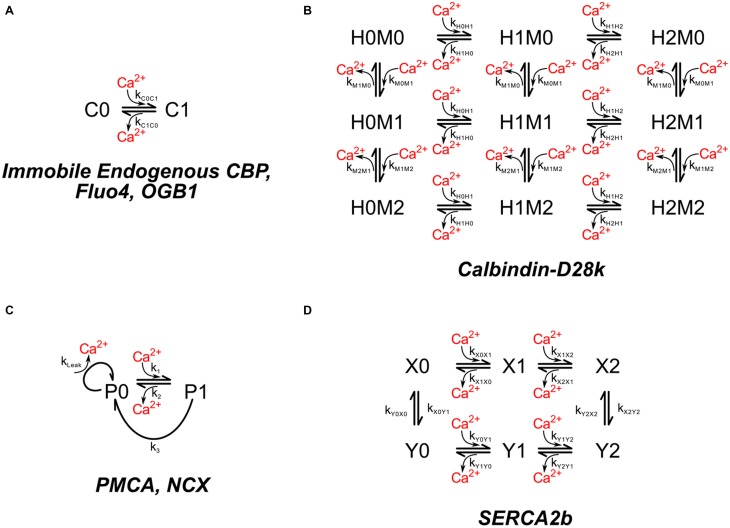
**Chemical kinetic schemes of endogenous Ca^2+^ buffer, calbindin-D28k, Ca^2+^ pumps, and Na^+^–Ca^2+^ exchanger. (A)** The interaction of Ca^2+^ with immobile endogenous CBPs was modeled as a simple first-order reversible reaction. Molecules of immobile endogenous CBPs were distributed throughout the volume of the dendritic and spine cytoplasm. **(B)** The mobile high affinity calcium buffer calbindin-D28k was included in the dendritic and spine cytoplasm. **(C)** Ca^2+^ pumps PMCA, and NCX were modeled with identical kinetic schemes but different rate constants. PMCA and NCX pumps were placed on the spines and dendritic shafts. **(D)** The SERCA pump was placed on ER membrane.

Ca^2+^ ion is buffered in the cytosol by binding to mobile and immobile “buffers.” The immobile buffers are considered to be immobile proteins with a mixture of low and high affinities for calcium (Neher and Augustine, 1992). To simulate these proteins we have used a single immobile buffer at a concentration of 78.7 μM with a *K*_*D*_ of 2.0 μM (Figure 3A). These values were chosen by calibrating the model so that it reproduces measurements of calcium fluxes after bAPs as described under Results Section. The concentration of immobile Ca^2+^ buffers in neurons have been estimated in the range of tens to a few 100 μM (Neher and Augustine, 1992; Maravall et al., 2000)

##### Calbindin

Calbindin is a major calcium buffering protein present in spines of hippocampal neurons. Its concentration in spines is debated. For simulations in which calbindin is included, we used 45 μM, the value reported for pyramidal neurons in area CA1 by Müller et al. (2005). We used the kinetic model for binding of Ca^2+^ to two high affinity sites and two medium affinity sites on calbindin published by Nagerl et al. (2000; Figure 3B; Table 1).

##### Fluorescent Ca^2+^ indicators

We calibrated parameters of the model by fitting the characteristic of simulated Ca^2+^ transients to measurements from Ca^2+^ transients in individual spines carried out with the use of the fluorescent Ca^2+^ indicators, Fluo-4 and Oregon Green 488-BAPTA (OGB-1; Sabatini et al., 2002). For the calibration process, a model of the appropriate fluorescent indicator was added to the dendritic cytosol as described under Results Section. The kinetic model for binding of Ca^2+^ by these indicators is shown in Figure 3A, and parameters are listed in Table 1. The diffusion constants were calculated from the Stokes/Einstein equation $D=\frac{kT}{6\pi \nu r}$. We set ν equal to $\frac{1}{3}$ the viscosity of water (0.001 $\frac{kg\times m}{s}$) to approximate the cytosol. To calculate *r* (the hydrodynamic radius of the diffusing particle), we first calculated the volume (*V*) from the molecular masses of Fluo4 and OGB1 (1100 and 1200 g/mol, respectively) and their densities (~1.4 g/cc). Both values were obtained from LifeTechnologies.com. We then assumed that $V=\frac{4\pi r^{3}}{3}$.

#### Removal of Ca^2+^ from the cytosol

Experiments with fluorescent Ca^2+^ indicators and pharmacological agents indicate that Ca^2+^ is removed from the spine cytosol by the combined action of three transmembrane proteins, Na^+^/Ca^2+^ exchangers (NCX's), smooth endoplasmic reticulum Ca^2+^ ATPases (SERCA's), and plasma membrane Ca^2+^ ATPases (PMCA's).

##### NCX's

The NCX's have a relatively low affinity for Ca^2+^ (*K*_*D*_ ~ 3 μM), but a large exchange capacity with turnover numbers sometimes as high as several thousand per second (Hilgemann et al., 1991; Carafoli et al., 2001). During calibration, the density of NCX's was set to 142 μm^−2^ on spines and 488 μm^−2^ on the shaft plasma membrane. These numbers are consistent with the finding of Lörincz et al. (2007) that shafts have a considerably higher density of NCXs than do spines. The kinetics of NCX were modeled by a Michaelis–Menten-like scheme (Figure 3C; Table 1). The turnover rate (k_3_) at which a pump with Ca^2+^ bound extrudes Ca^2+^ from the cell, was setat 600 s^−1^. The concentration of Ca^2+^ at which the pump operates at half maximum velocity (*K*_*M*_) was set at 3 μM (Blaustein and Lederer, 1999).

##### PMCA's

PMCA's, in contrast to NCX's, have a high affinity for Ca^2+^ (*K*_*D*_ < 0.5 μM) and use the energy of ATP hydrolysis to reduce the Ca^2+^ concentration to its resting level with a turnover rate of ~12 s^−1^ measured *in vitro* (Penheiter et al., 2003; Caride et al., 2007). We used the same kinetic scheme to model PMCA kinetics as for NCXs, but with parameters of 12 s^−1^ for turnover rate and 0.18 μM for K_M_ (Figure 3C; Table 1). The best fit to experimental measurements during the calibration (see Results and Discussion Sections), was obtained with a density of 998 μm^−2^ for PMCAs on the spine membrane and 488 μm^−2^ on the shaft plasma membrane.

##### SERCA's

About 19% of the spines in our sample contain SER. However, the ratio of spine SER membrane to spine plasma membrane is small, even in those spines. Nonetheless, we included SERCA pumps in the SER. The hippocampus expresses high levels of the SERCA2b isoform (Baba-Aissa et al., 1996), and only low levels of SERCA3 (Wuytack et al., 1995). We made the simplifying assumption that all of the SERCA pumps in CA1 pyramidal neurons are SERCA2B. SERCA pumps were placed on the SER membrane at a density of 1000 μm^−2^ (about half the density measured in RBL-2H3 mast cells by Means et al. (2006). The behavior of SERCA pumps is not fit well by the simple scheme that describes PMCA pumps (Figure 3C). Therefore, we modeled SERCA2b pumps according to a modification of the kinetic scheme from Higgins et al. (2006; Figure 3D; Table 1) for the SERCA pump at 37°C. We added individual Ca^2+^ binding events to the four state model in Higgins et al. (2006) to make a six state model, choosing a k_on_ of 1 × 10^8^ M^−1^ s^−1^ for binding of each Ca^2+^ to states X0 and X1 to satisfy the requirement for fast transition rates between X0, X1, and X2. We calculated the k_off_ values to maintain the *K*_*D*_'s specified in Higgins et al. (2006). The k_on_ rates for binding of Ca^2+^ to Y0 and Y1 were set to 1 × 10^5^ M^−1^ s^−1^ and the k_off_ rates were calculated according to the specified *K*_*D*_'s. These values allowed us to satisfy the Gibbs free energy constraints for binding and ATP hydrolysis as discussed in Higgins et al. (2006). We assumed a constant Ca^2+^ concentration of 60 μM in the ER lumen.

### Boundary conditions

Accurate modeling of a biochemical reaction–diffusion system requires appropriate treatment of the boundary conditions. We used a Neumann boundary condition; that is, we made the truncated ends of the two simulated dendritic segments reflective to Ca^2+^. This configuration causes Ca^2+^ flux to behave as if each dendritic segment were connected to a much greater length of dendrite, all seeing the same stimulus. Thus, when a back propagating action potential (bAP) activates VDCCs in a 5 μm length of dendrite, we assume that the electrical length constant of the dendrite would cause the bAP to activate VDCCs in the neighboring length of dendrite equally, causing Ca^2+^ ions to flow into and out of the segments at an equal rate.

The basal steady-state Ca^2+^ concentration was maintained at 100 nM by adjusting the leak rates of the PMCA's and NCX's (Figure 3C; Table 1). In addition to translocating Ca^2+^ out of the cell, PMCA's and NCX's allow a small leak of Ca^2+^ back into the cell. A leaky pump model is consistent with thermodynamic principles of reversibility and has significant advantages for calibration. If the rate of leaking and pumping is properly balanced in the rate constants of the kinetic scheme, then the resting [Ca^2+^] will be independent of pump density, and the proper resting [Ca^2+^] will be maintained with any number of pump molecules. The leaky pump model prevents the accidental introduction of standing gradients of Ca^2+^ or steady-state fluxes of Ca^2+^ along the length of the resting dendrite or between the spines and dendritic shaft. In contrast, standing gradients and fluxes would be virtually impossible to eliminate if the pumps and leaks were implemented as separate proteins. We used Michaelis–Menten equations to calculate the leak rate as follows. We assumed that the pump has an average basal extrusion rate (*r*_*ex*_) at 100 nM Ca^2+^ that is related to the fraction of pumps with bound Ca^2+^ (*f*_*b*_) at that concentration: $r_{ex}=f_{b}{}^{*}k_{3}$. The fraction of pumps with bound Ca^2+^, both at rest and after a stimulus, depends on the Ca^2+^ concentration. The quasi-steady-state assumption (Briggs and Haldane, 1925) yields the relations:

(3) $$\left[P_{Ca^{2+}}\right]=\frac{{\left[P\right]}_{tot}\left[Ca^{2+}\right]}{K_{M}+\left[Ca^{2+}\right]},$$

and

(4) $$K_{m}=\frac{k_{2}+k_{3}}{k_{1}},$$

where $\left[P_{Ca^{2+}}\right]$ is the number of pumps with Ca^2+^ bound.

(5) $$f_{b}=\frac{\left[P_{Ca^{2+}}\right]}{{\left[P\right]}_{tot}},$$

therefore, substituting for *K*_*M*_ and multiplying by *k*_1_ gives the relation:

(6) $$f_{b}=\frac{k_{1}\left[Ca^{2+}\right]}{k_{2}+k_{3}+k_{1}\left[Ca^{2+}\right]},$$

which relates *f*_*b*_ to [Ca^2+^].

Thus, we calculated *f*_*b*_ and *r*_*ex*_ at resting [Ca^2+^] = 100 nM from the rate constants for PMCA's and NCX's in Table 1. We then set their leak rates equal to the calculated basal *r*_*ex*_ to produce a resting steady-state [Ca^2+^] of 100 nM. We set the Ca^2+^ leak rate for PMCA's at 4.3 s^−1^ and for NCX's at 19.4 s^−1^ (Table 1). These rates produced a steady-state basal [Ca^2+^] of 100 nM in simulations in which no stimulus was applied.

### Modeling of fluorescence experiments

To simulate experimental results obtained with Ca^2+^ sensitive fluorescent dyes, we added 20 μM Fluo4, 50 μM Oregon Green Bapta 1 (OGB1), or 100 μM OGB1 to dendrites, as appropriate (Figure 3A; Table 1); and then simulated interaction of Ca^2+^ with the indicator with a time step of 0.1 μs during a simulated bAP stimulus, using MCell's adaptive time stepping. We directly recorded the amount of Ca^2+^ bound to indicator, the amount of unbound indicator, and the amount of free Ca^2+^ in the spines and shafts every 100 μs. Thus, we did not need to simulate the resulting fluorescence to obtain information about Ca^2+^ transients. To directly compare our simulation results to the experimental results of Sabatini et al. (2002), which they calculated using the method of Maravall et al. (2000), we estimated the Ca^2+^ transient by assuming that Ca^2+^ reaches rapid equilibrium with the fluorescent dye. At equilibrium,

(7) $$\left[Ca^{2+}\right]=\frac{K_{D}\left[B\right]}{\left[U\right]},$$

where *K*_*D*_ is the dissociation constant of Ca^2+^ from the dye, [*B*] is the concentration of dye bound to Ca^2+^, and [*U*] is the concentration of unbound dye.

Therefore, the estimated Ca^2+^ transient at each time step, $\Delta {\left[Ca^{2+}\right]}_{estim}$, will be:

(8) $$\Delta {\left[Ca^{2+}\right]}_{estim}=\left(\frac{K_{D}\left[B\right]}{\left[U\right]}\right)-{\left[Ca^{2+}\right]}_{0},$$

where ${\left[Ca^{2+}\right]}_{0}$ is the steady state resting concentration of Ca^2+^ with no stimulus (i.e., 100 nM). To accurately simulate the data of Sabatini et al. (2002), we lowpass filtered the simulated transients ($\Delta {\left[Ca^{2+}\right]}_{estim}$) in the spine head and adjacent shaft (captured with a resolution of 1 μs) with a 250 Hz four-pole Bessel lowpass filter and sampled at 500 Hz to correspond with the experimental measurements.

### Statistics

One hundred individual simulations, each with a new random seed, were carried out with each set of parameters. Ca^2+^ transients were recorded in the large spines (≥0.05 fl in volume) on the smaller diameter dendrite (seven spines) for calibration and in all spines on the larger diameter dendrite for later tests. The individual simulations were averaged for each spine, and then the means from each spine were averaged and the standard error of the mean was determined. This procedure closely recapitulates the statistical analysis of the experimental data (see Section Results). The random placement of membrane molecules for each simulation with a new random seed introduces more variability into simulations in each spine than would be observed in repeated scans of the same spine in an experiment, because it introduces stochastic variation of channel numbers within each individual simulated spine. Thus, the simulations mimic sampling of 100 spines with the same shape and size, but slightly different numbers and arrangements of proteins.

## Results

### Morphological analysis

**Figure 4:**
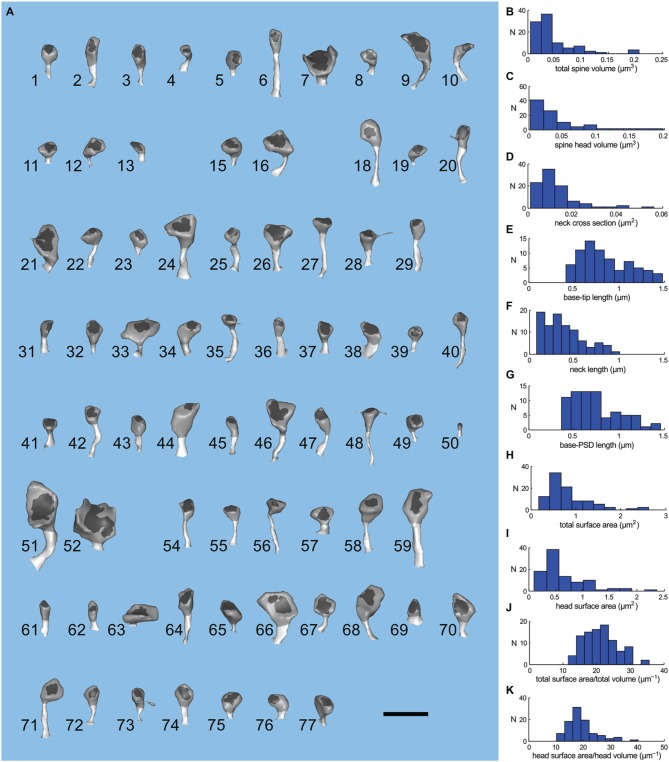
**Geometry of spines of the large apical dendrite. (A)** Visual index of the spines of the large apical dendrite. Spines have been oriented to view both head and neck. The PSD region is dark gray; spine head, medium gray; and spine neck, light gray. The spines are numbered 1–77 and have not been sorted. Five spines (numbers 14, 17, 30, 53, and 60) were omitted from the simulations because they were clipped by the boundary of the reconstructed volume. Thus, the apical dendritic segment contains 72 intact spines. The scale bar is 1 μm. **(B–K)** Distribution of geometric parameters of the spines. **(B)** Total spine volume. **(C)** Spine head volume. The average spine head volume in the sample was 0.03 μm^3^. **(D)** Spine neck cross sectional area. **(E)** Base-tip length. The average spine length from the base of the spine to the apex of the head was 0.85 μm. **(F)** Spine neck length. **(G)** Base to PSD length. **(H**) Total surface area. **(I**) Head surface area. **(J)** Ratio of total spine surface area to total spine volume. **(K)** Ratio of head surface area to head volume.

To compare the range of shapes and sizes of spines in the reconstructed neuropil to those reported previously in fixed hippocampal tissue, we measured several morphological parameters of the 72 spines (Figure 4A) on the larger reconstructed dendrite (Figures 4B–K). Spine head volumes ranged from 0.004 to 0.2 fl; base-to-tip lengths of spines from 0.48 to 1.45 μm; diameters of spine necks from 0.04 to 0.5 μm; and spine neck lengths from 0.08 to 1.1 μm. The average volume of spine heads was 0.03 μm^3^ (0.03 fl) and the average base-tip length of spines was 0.85 μm. These measurements are consistent with those of Harris and Stevens (1989) and as reviewed in Sorra and Harris (2000). The measurements were made after the extracellular volume fraction of the reconstruction was expanded from 8 to 20% to account for shrinkage artifacts during fixation (Kinney, 2009; Kinney et al., 2013). Thus, the good agreement with previous measurements shows that no significant distortions of the geometry were introduced by our correction methods.

### Calibration and parameter sensitivities

Although most of the kinetic parameters listed in Table 1 are reasonably well-constrained by values in the literature; the concentrations of some of the proteins within neurons are not known as precisely as others because they have been estimated based upon relatively low resolution data. Thus, to bring the Ca^2+^ transients predicted by the model into agreement with experiments of Sabatini et al. (2002), we adjusted nine parameters within reasonable previously measured physiological ranges, seven concentration parameters and the k_on_ and k_off_ of the endogenous buffer. The concentration parameters that we adjusted were: the densities of the PMCA, NCX, and VDCCs on spine and on shaft membranes; and the concentration of the immobile endogenous CBPs, which represent the sum of endogenous Ca^2+^-binding proteins (Table 2). In adjusting the concentrations of the PMCA and NCX pumps, we used an additional experimental constraint from Scheuss et al. (2006), who reported that PMCAs and NCXs each remove approximately the same amount of Ca^2+^ in response to a bAP. Thus, we adjusted the ratio of PMCA to NCX pumps such that they would extrude equal amounts of Ca^2+^ in spines of the size measured by Sabatini et al. (2002). This constraint reduced the number of free parameters to seven. The range over which each parameter was tested by manual trial and error to obtain initial preliminary best fit values is indicated in Table 2. The data upon which the ranges are based is discussed for each protein in the Methods Section under Proteins.

**Table 2:** **Parameter sensitivity in four dimensional measurement space**.

**Parameter**	*z* at 1.5*p	*z* at 0.9*p	**Dimensionless sensitivity (Δ*z*/0.6)**	**Δ*p* for +1σ change (Δ*p*/Δ*z*)**	**Range of exploration**
CBP on rate	16.5	10.9	9.3	16.1 μM^−1^s^−1^	50–800 μM^−1^s^−1^
CBP off rate	9.5	13.7	–7.0	–45.5 s^−1^	100–780 s^−1^
CBP concentration	19.4	10.7	14.5	3.3 μM	0–300 μM
VDCC density spine	13.8	12.1	2.8	0.256 μm^−2^	1.08–9.0 μm^−2^
VDCC density shaft	21.2	16.0	8.7	0.185 μm^−2^	1.8–9.0 μm^−2^
Pump density spine	13.6	12.7	1.5	57.2 μm^−2^ NCX and 400 μm^−2^ PMCA	35–284 μm^−1^ NCX and 500–4000 μm^−1^ PMCA
Pump density shaft	27.0	14.4	21.1	14.2 μm^−2^ NCX and 14.2 μm^−2^ PMCA	125–1000 μm^−2^ NCX and 250–2000 μm^−2^ PMCA

Values for z were calculated as described in Results Section (Equation 13). Values for p and Δp are based on the parameter values obtained for the preliminary best fit, as described in Results Section. Range of exploration indicates the range of parameter values that were used during the search for the preliminary best fit. Note that the number of free parameters was reduced to seven from nine because the ratio of NCX and PMCA pumps was constrained as described under “Calibration and parameter sensitivities.”

**Figure 5:**
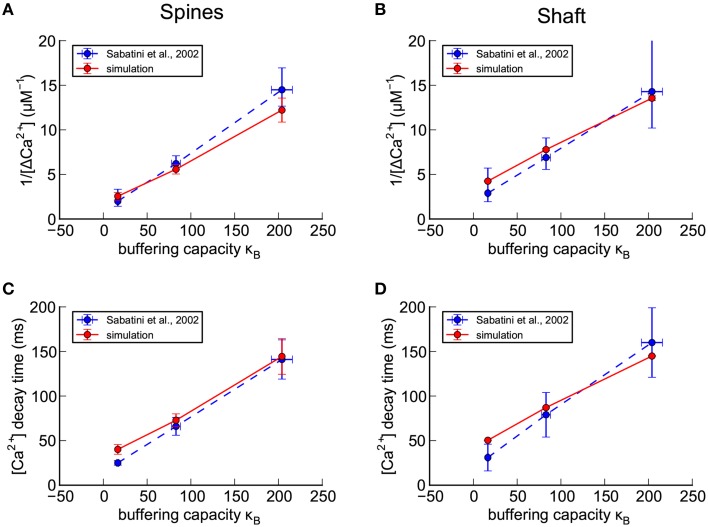
**Calibration of nine parameters in spines and shafts of the small branch spine. (A–D)** Confirmation of calibration of the shape of a Ca^2+^ transient in response to a bAP in the absence of calbindin (see Methods Section). For comparison with experimental results of Sabatini et al. (2002; shown as blue dashed lines), concentrations of exogenous fluorescent calcium indicators in the model were set to 20 μM Fluo4, 50 μM OGB1, or 100 μM OGB1. The simulated results for the best calibrated parameters, averaged for seven spines with volumes ≥ 0.05 fl, are shown as red lines. **(A)** For each concentration of indicator, the reciprocal of the averaged peaks of the calcium transients in the spines, 1/Δ[Ca^2+^], is plotted against the buffering capacity in the presence of indicator (κ_*B*_) calculated as in Sabatini et al. (2002). The Δ[Ca^2+^], extrapolated to zero indicator, was in the range 0.5 to 1.0 μM (reciprocal of y-axis intercept) with κ_*e*_ in the range 16–50 (x-axis intercept). **(B)** The reciprocal of the averaged peaks of the calcium transients in the dendritic shaft is plotted against buffering capacity. The Δ[Ca^2+^], extrapolated to zero indicator, was 0.3 μM with κ_*e*_ = 62. **(C)** The averaged time constant of calcium decay in the spines is plotted against calcium buffering capacity. The calcium decay time constant, extrapolated to zero indicator in the spines was in the range 20–40 ms (y-axis intercept) with κ_*e*_ in the range 30–80 (x-axis intercept). **(D)** The averaged time constant of calcium decay in the dendritic shafts was plotted against buffering capacity. The calcium decay time constant, extrapolated to zero indicator in the dendritic shafts was 42 ms with κ_*e*_ = 75.

In their experiments, Sabatini et al. (2002) placed whole-cell clamps on the somas of CA1 pyramidal neurons and perfused them with three different fluorescent Ca^2+^ indicators at different concentrations to generate data from spines with a gradient of Ca^2+^ buffering capacities when buffering by the dyes is included. During the perfusion, as indicator diffused throughout the neuron, we assume that calbindin was washed out of spines because its size and diffusion constant are similar to those of the fluorescent indicators. Sabatini et al. then used a two-photon laser-scanning microscope, scanning at 500 Hz, to record the fluorescent transients in spines and the adjacent dendritic shafts in response to a bAP stimulus triggered by injecting current through the patch pipette. They state that Ca^2+^ transients were measured on small apical dendrites less than 2 μm in diameter and on spines in which the fluorescent signal could be reliably detected. Their data was gathered from 7 to 14 spines at four concentrations of indicator with six scans of each spine during which a single bAP was delivered. The fluorescent transients were converted into an estimate of the size of the Ca^2+^ transient, Δ[Ca^2+^]_estim_, using a previously published method (Maravall et al., 2000). Transients calculated from the six scans were then averaged for each spine, and then the means from each spine were averaged and the standard error of that mean was calculated. They determined the resting Ca^2+^ concentration in spines and shafts, the change in Ca^2+^ in response to a backpropagating action potential, and the decay time constant of the Ca^2+^ transient (τ), at each concentration of Ca^2+^ indicator. The endogenous buffering capacity, κ_*e*_, of spines and shafts was then calculated by the method of Neher and Augustine (1992) in which the relationship of the size of the Ca^2+^ transient to the buffering capacity of the Ca^2+^ indicators is extrapolated to zero indicator concentration (see Figure 5).

To simulate the Sabatini et al. (2002) experiments, we set the calbindin concentration in our model to zero, and added different Ca^2+^-sensing dyes to the model as described under Methods Section. We recorded the amount of Ca^2+^ bound to each indicator over time during a simulated bAP stimulus. For calibration, we used simulation data collected from spines on the smaller dendrite (shaft diameter 0.8 μm, Figure 1E) because its dimensions are closest to those in which Sabatini et al. (2002) made their measurements. In addition, we used only spines with volumes of 0.05 fl or larger (seven spines) because we reasoned that only the larger spines would have provided readily detectable and reliable fluorescence signals in an experimental setting.

We set four target values from the Sabatini et al. data, for each of the three different Ca^2+^ indicator concentrations: 1/peak Δ[Ca^2+^] and decay time (τ) for transients in the spine head, and the same two values for transients in the shaft (Figure 5). We then determined these four values from each of our simulations, as described in Methods Section, for each indicator concentration and parameter set.

To obtain preliminary values for the seven parameters that best fit the target values, we made manual systematic iterative adjustments of the densities of PMCAs, NCXs, and VDCCs, in the membranes, varying their values within the boundaries of estimates of their concentrations in the literature (see Methods Section and Table 2). We estimated the ratio of PMCA and NCX pumps that would remove equal amounts of Ca^2+^ from the spine head, as found in Sabatini et al. (2002), by calculating the mean concentration of Ca^2+^ during a typical transient and then using the K_M_'s and turnover numbers of the two pumps to calculate the ratio of the pumps that would be required for each to remove an equal amount of Ca^2+^ at that concentration. The pumps were then kept at that ratio in both spine and shaft membranes, and the numbers of the pumps in spines and shafts were adjusted to keep this ratio constant during the trials to find preliminary best fit parameters. In other words, the densities of the two pumps in the spine head and in the shaft were each treated as one parameter, reducing the number of free parameters from nine to seven (Table 2). Before proceeding to the refinement stage described below, the ratio of their densities was readjusted slightly so that each of the pumps removed equal amounts of Ca^2+^ from both the shafts and the large spine heads. The concentration and Ca^2+^-binding rates of the immobile endogenous CBP in the cytoplasm were also adjusted until a reasonably good fit was obtained to the experimental plots of total buffer capacity, κ_*B*_, against the amplitudes and decay times of the Ca^2+^ transients, in the presence of three different concentrations of fluorescent sensors (Figure 5).

We next refined the local fit and determined approximate parameter sensitivities using a form of Principal Component Analysis as follows. First, we constructed three 4 × 4 covariance matrices (Σ, Equation 9) for the three sets of four output values determined from the simulation data we collected from the seven spines. The calculations are summarized in Equations (10–12), where *n* is the number of spines observed (*n* = 7), *x*_*i*_ and *y*_*i*_ are the *i*th observation of output values *x* or *y*, respectively. Likewise, μ_*x*_ and μ_*y*_ are the mean output value for *x* or *y*, across *n* = seven spines.

(9) $$\sum_{xy}=\left[\begin{array}{cccc} var_{1} & cov_{12} & cov_{13} & cov_{14}\\ cov_{21} & var_{2} & cov_{23} & cov_{24}\\ cov_{31} & cov_{32} & var_{3} & cov_{34}\\ cov_{41} & cov_{42} & cov_{43} & var_{4} \end{array}\right],$$

where

(10) $$var_{x}=\frac{1}{n}\sum_{i\;=\;1}^{n}{\left(x_{i}-\mu_{x}\right)}^{2},$$

(11) $$cov_{xy}=\frac{1}{n}\sum_{i\;=\;1}^{n}\left(x_{i}-\mu_{x})(y_{i}-\mu_{y}\right),$$

and

(12) $$\mu_{x}=\frac{1}{n}\sum_{i\;=\;1}^{n}x_{i}.$$

We then averaged the three matrices together, element by element, to create an average covariance matrix. For this calculation, it would be ideal to use the experimental covariances. However, because we don't have access to the raw experimental data, we instead computed the average of the three covariance matrices from the simulation results. To the extent that we have chosen the correct arrangement of proteins for the simulations, this matrix should be similar to an experimental covariance matrix. The averaging reduces the dimensionality of the aggregate “output value space” from 12D (3 × 4D), which would be underdetermined by our seven variable parameters, to 4D, which is, instead, overdetermined by the seven variable parameters. This method is adequate for estimating parameter sensitivies and checking the goodness of fit, given the boundaries on the variable parameters, and the inherent noisiness of the original experimental measurements of Ca^2+^ transients by fluorescence methods.

We know from computational statistics and Principle Component Analysis that the four eigenvalues of this covariance matrix are the interdependent variances of each of the four output measurements in the direction of the eigenvectors (axes of the principle components). Thus, the square root of the eigenvalues equals 1 standard deviation (i.e., 1 sigma) in the direction of its associated eigenvector. We used the eigenvalues and eigenvectors to refine the fit and determine the parameter sensitivities by the following method. We ran 14 simulations in which each of the seven parameters was varied independently either increasing it to 1.5 times its preliminary best fit value, or decreasing it to 0.9 times its preliminary best fit value (14 groups of parameters). These increments were chosen so that the resulting change in the output value was large enough not to be occluded by the Monte–Carlo trial-to-trial variability, but small enough to ensure rough linearity between the change in output value and the change in the parameter value. These simulations were run for each of the three indicator concentrations. For each set of parameters, the four output values were calculated for each of the seven spines, averaging 100 simulation runs. Then the mean output values were averaged over the seven spines to obtain three sets of four averaged output values for each set of parameters at the three indicator concentrations. The three sets of four values were then averaged to obtain a single set of four values for each of the 14 sets of parameters. The target (experimental) values were represented as a point in four-space and each group of output values for the 14 sets of parameters was also represented as a point in four-space. The radial distances from the point in four-space representing the target (experimental) values to the point in four-space for each set of parameters was then calculated, and normalized by the square root of the corresponding eigenvalue (i.e., the standard deviation of the corresponding principal component) (Note that, after normalizing the radial distance we obtain, *z*, the Z-score in four dimensions). To do this calculation, the origins of the four eigenvectors with lengths scaled to 1 sigma, as derived from the corresponding eigenvalues, were placed at the four-dimensional point representing the four target experimental values. Then the distances (*z*) between the four experimental target values and the corresponding output values for the 14 sets of parameters was calculated by projecting the 14 four-dimensional points onto the four scaled eigenvectors. The ratio of the length of each projection to the length of the eigenvectors is a measure that represents the number of standard deviations, or Z-score, of one of the simulated values from the target value (i.e., 1/Δ[Ca^2+^] in the spine head or shaft, or decay time (τ) in the spine head or shaft). This operation projects, or transforms, the four original values into a new four-dimensional measurement space where the four transformed values are the coordinates of a point, *m*, whose distance, *z* (normalized by the standard deviation in each dimension) from the point representing the target value is given by the equation:

(13) $$z=\sqrt{\sum_{i\;=\;1}^{4}{\left(m_{i}^{s}-m_{i}^{t}\right)}^{2}},$$

where the subscript, *i*, is the *i*^th^ dimension of point *m*, the superscript *s* indicates the transformed simulated value, and superscript *t* indicates the transformed target experimental value. The change in *z*, Δ*z*, over the fractional change in parameter value *p*, $\frac{\Delta p}{p}$, is $\frac{\Delta z\cdot p}{\Delta p}$ = $\frac{\Delta z}{0.6}$ for each parameter, where Δ*p* = 1.5*p* − 0.9*p* or 0.6 × the preliminary best fit value. In the language of Principal Component Analysis, this calculation means that the difference vector between simulated and target measurements was projected onto the four principal components, and the resulting difference vector components were normalized by the standard deviations of these principal components.

We expressed the parameter sensitivity in Table 2 in two different ways. The first measure, $\frac{\Delta z}{0.6}$, is a dimensionless measure that reflects the change in goodness of fit to the experimental target that results from changing *p* from 0.9^*^p to 1.5^*^p. The second measure, $\frac{\Delta p}{\Delta z}$ is a measure that indicates the change in *p* necessary to produce a change of +1σ in the goodness of fit to the experimental target. The values of $\frac{\Delta z}{0.6}$ in Table 2 indicate that VDCC density and pump density in the spine (2.8 and 1.5, respectively) are the least sensitive parameters and that the CBP concentration and pump density in the shaft (14.5 and 21.1, respectively) are the most sensitive parameters affecting the goodness of fit. It is important to note that *z* measures the goodness of fit in all four dimensions simultaneously. Furthermore, the goodness of fit is weighted equally by the variance of the simulation data in each of the four dimensions. This means that the simulation measurements that have smaller variances will affect the fit more sensitively than those with larger variances. It is evident in Figures 5B,D, that the variances of simulation measurements in the shaft are smaller than those in the spine. For this reason, the fit is more sensitive to parameters in the shaft than the corresponding parameters in the spine.

The column headed “Range of exploration” in Table 2 shows the range of values explored for each parameter during the manual search for the preliminary best fit. These ranges are consistent with the range of experimental measurements, as discussed in Methods Section. Though we cannot guarantee with certainty that other combinations of parameters will not fit as well as the values shown in Table 3, we found no such combinations within the indicated ranges of values.

**Table 3:** **Calibrated parameters**.

**Parameter**	**Calibrated value**	**Range in the literature**
**PMCA PUMPS**		
Density in spine membrane	998 μm^−2^	~300 μm^−2^ in neuronal cells, if total membrane protein density is taken as ~30,000 μm^−2^ and PMCA as 1% (Stauffer et al., 1995). This number is an estimate.
Density in shaft membrane	488 μm^−2^	
**NCX PUMPS**		
Density in spine membrane	143 μm^−2^	Little information on absolute density. Lörincz et al. (2007) report a ratio of ~7:1 for density on shaft vs. spine. Our best fit ratio is 3.4:1.
Density in shaft membrane	488 μm^−2^	
Ratio of Ca^2+^ pumped by PMCAs and NCXs	1:1	Ratio of 1:1 determined by Scheuss et al. (2006).
**VDCCs**		
Density in spine membrane	1.2 μm^−2^	Magee and Johnston (1995a) estimated a density of 1–2 μm^−2^for R-type Ca^2+^ channels on dendrites of CA1 pyramidal neurons. In contrast, using noise analysis, Sabatini and Svoboda (2000) predicted as high as 20 μm^−2^in spines.
Density in shaft membrane	2.67 μm^−2^	
**IMMOBILE CBPs**		
Concentration in cytosol	78.7 μM	
k_on_	2.47 × 10^8^ M^−1^s^−1^	
k_off_	524 s^−1^	

*Nine parameters were calibrated to fit the experimental data in Sabatini et al. (2002) as described in the Results Section. The ratio of 1:1 for the amount of Ca^2+^ pumped by PMCAs and NCXs was set at 1:1 to match data in Scheuss et al. (2006), reducing the free parameters to seven*.

To further improve the fit, we re-adjusted each parameter in the initial best-fit parameter set by an appropriate amount to decrease the final distance *z*, based on its $\frac{\Delta z\cdot p}{\Delta p}$. We assumed linearity of Δ*z*over the change in parameter values (Δ*p* = 0.6*p*) and extrapolated to minimum *z*. The output values calculated from the simulations showed a small improvement in fit and are plotted in Figures 5A–D. Additional adjustments of the parameters didn't significantly improve the overall fit. Table 3 shows the final parameters. For comparison, it also shows the corresponding experimental estimates of the parameters available in the literature.

The simulations predicted endogenous buffering capacities (κ_*e*_) in the same range as reported by Sabatini et al. (2002) with values from 16 to 80 when the concentration of indicator was extrapolated to zero (see Methods Section and Figures 5A–D). The decay time constant for the Δ[Ca^2+^]_estim_ transient was 42 ms in the dendrites and 20–40 ms in the spines when extrapolated to zero indicator, again in the same range reported by Sabatini et al. (2002). The extrapolated peak Δ[Ca^2+^]_estim_ during the bAP was 0.3 μM in the dendrites and 0.5–1.0 μM in the spines, also in the range reported by Sabatini et al.

### Relationship of buffering capacity to amplitude and decay time of Δ[ca^2+^]_estim_ in the large dendrite

**Figure 6:**
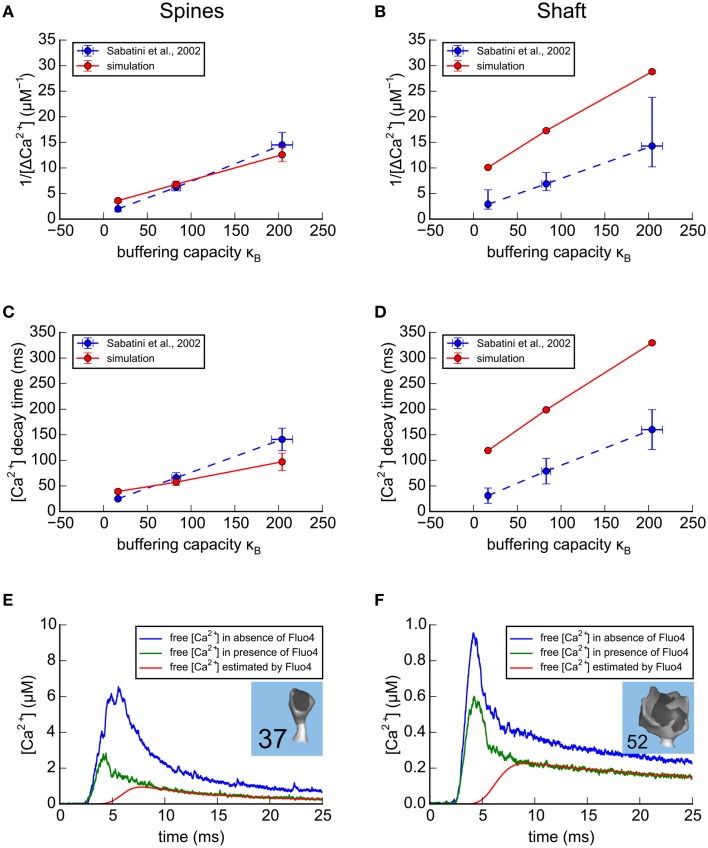
**Results of simulations in the spines and shafts of the large apical dendrite using parameters optimized for the small branch dendrite. (A–D)** Simulation of Ca^2+^ transients in response to a bAP in the absence of calbindin. As in Figure 5, for comparison with experimental results of Sabatini et al. (2002; shown as blue dashed lines), concentrations of exogenous fluorescent Ca^2+^ indicators in the model were set to 20 μM Fluo4, 50 μM OGB1, or 100 μM OGB1. The simulated results in the large apical dendrite, averaged for the 13 spines with volumes ≥0.05 fl are shown as red lines. **(A)** For each concentration of indicator, the reciprocal of the averaged peaks of the Ca^2+^ transients in the spines of the large apical dendrite, 1/Δ[Ca^2+^], is plotted against the buffering capacity in the presence of indicator (κ_*B*_) calculated as in Sabatini et al. (2002). The Δ[Ca^2+^], extrapolated to zero indicator, was in the range 0.3–0.6 μM (reciprocal of y-axis intercept) with κ_*e*_ in the range 40–75 (x-axis intercept). **(B)** The reciprocal of the averaged peaks of the Ca^2+^ transients in the dendritic shaft is plotted against buffering capacity. The Δ[Ca^2+^], extrapolated to zero indicator, was 0.12 μM with κ_*e*_ = 75. **(C)** The averaged time constant of Ca^2+^ decay in the spines is plotted against Ca^2+^ buffering capacity. The Ca^2+^ decay time constant, extrapolated to zero indicator in the spines was 25 to 40 ms (y-axis intercept) with κ_*e*_in the range 60–120 (x-axis intercept). **(D)** The averaged time constant of Ca^2+^ decay in the dendritic shafts was plotted against buffering capacity. The Ca^2+^ decay time constant, extrapolated to zero indicator in the dendritic shafts was 100 ms with κ_*e*_ = 80. **(E,F)** Ca^2+^ transients in two spines of the calibrated model, in response to a bAP. Simulations were run in the presence and absence of 20 μM Fluo4 in a median size spine (**E**, spine #37, average of 32 trials) and in a large-size spine (**F**, spine #52, average of 59 trials). The average Ca^2+^ transients that would be estimated from the fluorescence of 20 μM Fluo4 are shown in red. The average Ca^2+^ transients, measured in the simulation by counting actual free Ca^2+^, are shown in the presence of Fluo4 (green), and in its absence (blue).

After we calibrated the model to match experimental data using the small dendrite, we used the same parameters to simulate Ca^2+^ transients for large spines on the large dendrite (13 spines ≥ 0.05 fl, shaft diameter ~2.1 μm (Figures 6A–D). The most striking difference between Ca^2+^ transients in the two dendrites is seen in the shafts. For each concentration of indicator, the simulated transient in the large shaft has a smaller amplitude (e.g., 1/Δ[Ca^2+^] is larger) than in the small shaft, and the decay time is longer, reflecting the smaller surface to volume ratio of the shaft with the larger diameter.

### Effect of the presence of fluorescent indicators on the free Ca^2+^ transient

One advantage of this model is that after it is calibrated to agree with experiments that were performed with Ca^2+^ indicators, they can be removed from the simulations to explore Ca^2+^ dynamics in the absence of indicators. We know that exogenous indicators alter the free Ca^2+^ transient, but their effect can't be measured by direct experiments. We used the calibrated model to simulate and compare free Ca^2+^ transients in a small (Figure 6E) and a large (Figure 6F) spine in response to a bAP as estimated from fluorescence measurements by the method of Maravall (red), and as recorded in the simulation in the presence (green) and absence (blue) of Fluo4. The peak of the free Ca^2+^ estimated from fluorescence is much smaller than the free Ca^2+^ transients recorded during the simulations, either in the presence or absence of Fluo4. This is because the response of the indicator is too slow to follow the rapid initial phase of the transient. Note that the indicator does accurately follow the slow decay phase of the transient when the free [Ca^2+^] is changing slowly enough that the indicator is able to track it.

### Responses to EPSP, bAP, and EPSP+bAP stimuli in a median-size spine

**Figure 7:**
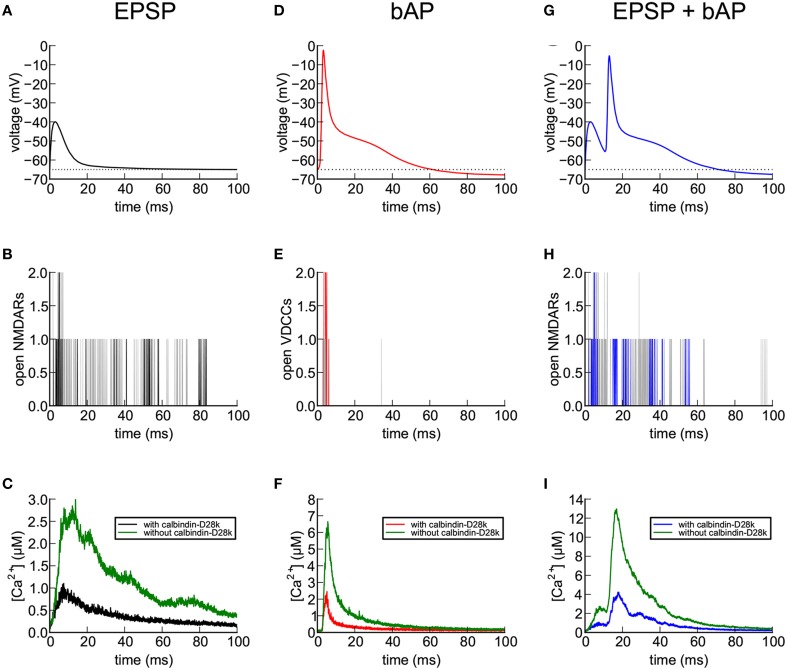
**Calcium entry into spines in response to EPSP, bAP, and EPSP+bAP stimuli**. Unless otherwise stated, the dendrite contained 45 μM calbindin-D28k and the signals are those recorded in spine #37. **(A)** Depolarization of the spine head following an EPSP stimulus. The voltage signal was generated from a NEURON simulation, as described in Methods Section. **(B)** NMDARs flickered between open and closed states during an EPSP. The black trace shows a single trial. Gray traces are superposition of five additional trials. **(C)** Ca^2+^ transients in the spine head in response to a single EPSP stimulus averaged over 56 trials in the presence (black) and absence (green) of calbindin-D28k. **(D)** Depolarization of the spine head following a bAP stimulus. The voltage signal was generated from a NEURON simulation as described in Methods Section). **(E)** VDCCs flickered between open and closed states during the bAP. The red trace shows a single trial. Gray traces are superposition of five additional trials. **(F)** Ca^2+^ transients in the spine head in response to a single bAP stimulus averaged over 32 trials in the presence (red) and absence (green) of calbindin-D28k. **(G)** Depolarization of the spine head following an EPSP+bAP stimulus. The voltage signal was generated from a NEURON simulation as described in Methods Section. **(H)** NMDARs flickered between open and closed states during an EPSP+bAP. The blue trace shows a single trial. Gray traces are superposition of five additional trials. **(I)** Ca^2+^ transients in the spine head in response to a single EPSP+bAP stimulus averaged over 86 trials in the presence (blue) and absence (green) of calbindin-D28k.

We examined simulated Ca^2+^ transients in the absence of indicator in more detail in a single median-size spine (#37), in response to three different stimuli; an EPSP alone, a bAP alone, and an EPSP preceding a bAP by 10 ms (EPSP+bAP; Figure 7). During the stimuli that included an EPSP, 1500 molecules of glutamate was released from a single site and bound to and activated AMPARs and NMDARs. We previously found that release of 1500 molecules of glutamate produces activation that most closely matches experimental estimates (Keller et al., 2008). The EPSP voltage was simulated as described under “Stimuli” in Methods Section.

During a single EPSP (Figure 7A), activated NMDARs are mostly blocked by Mg^2+^. Thus, in the simulation, only a few flickered open stochastically (Figure 7B). The size of the EPSP (25 mV) is the average size for hippocampal CA1 pyramidal neurons, calculated by Harnett et al. (2012). Many of the EPSP trials in this simulation resulted in no Ca^2+^ influx at all because of the small number of activated and unblocked NMDARs. We computed the average of Ca^2+^ transients during the successful trials both in the presence (black) and absence (green) of calbindin (Figure 7C). In the presence of calbindin, successful trials produced a slow rising phase to a peak amplitude of about 1 μM, followed by a slow decay. In the absence of calbindin, the average peak amplitude was about 2.6 μM. Thus, the principal effect of calbindin on the Ca^2+^ transient is to reduce its overall magnitude. The noisiness in the averaged trace of the Ca^2+^ transient in Figure 7C is a result of stochastic opening and closing of the NMDARs and binding and unbinding of Ca^2+^ to the CBPs and pumps.

During a bAP (Figure 7D), the depolarization of the spine head caused the small number of VDCCs to flicker open and closed stochastically (Figure 7E) and Ca^2+^ flowed into the spine head through the open channels (Figure 7F). As with the EPSP stimulus, many trials with the bAP stimulus failed to open any VDCC channels and no Ca^2+^ influx occurred. We averaged the successful Ca^2+^ responses to a bAP and observed a rapid rising phase to a peak amplitude of about 2.2 μM in the presence of calbindin, followed by a rapid decay, and a peak of 6.5 μM in its absence. During the sustained influx of Ca^2+^, the rate of influx exceeded the rate at which internal Ca^2+^-binding proteins could bind the Ca^2+^, resulting in a rapid rise of free Ca^2+^ levels. When the Ca^2+^ influx stopped, the Ca^2+^-binding proteins and free Ca^2+^ rapidly reached a quasi-equilibrium. The Ca^2+^ was then removed from the spine by the Ca^2+^ pumps and by diffusion through the spine neck.

After a simulated EPSP+bAP stimulus (Figure 7G), only zero, one, or (rarely) two NMDARs became doubly bound by glutamate immediately after its release. Those that did cycled stochastically between closed and open states for much longer than VDCCs (Figure 7H). Because the Mg^2+^ block was relieved by the bAP, a large Ca^2+^ influx was observed in the spine head, followed by decay to baseline (Figure 7I). In the presence of calbindin (blue), the average amplitude of the Ca^2+^ transients during trials that resulted in Ca^2+^ influx was 4.0 μM and the time to decay to 10% of the peak was 65 ms. In the absence of calbindin (green), the peak amplitude was nearly 13 μM and the time to decay to 10% of the peak was 50 ms.

**Figure 8:**
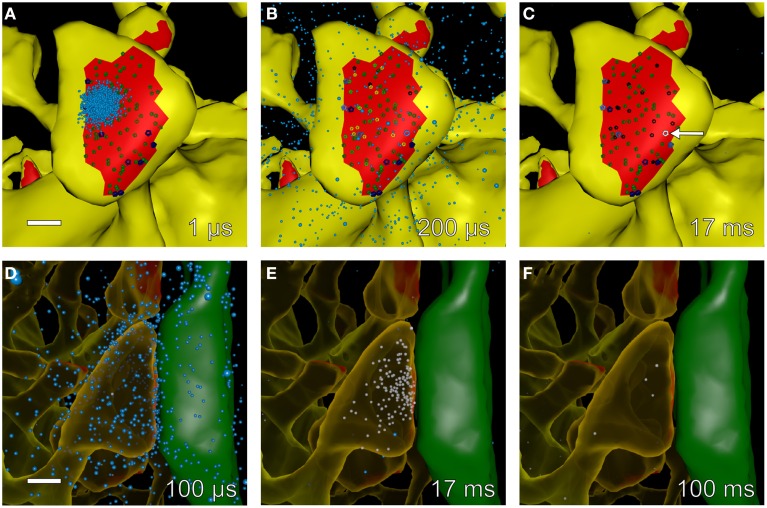
**Snapshots of simulated glutamate release and postsynaptic calcium dynamics at different times during an EPSP+bAP stimulus**. We used Cell Blender (see Methods Section) to visualize simulated glutamate release and calcium entry into spine #37 (see Figure 4A) during an EPSP+bAP stimulus. All other components of the neuropil were present in the simulation but are not displayed in the visualization. All scale bars are 0.25 μm. **(A–C)** Time-lapse sequence of glutamate (cyan dots) released onto a dendritic spine with unbound AMPARs (smaller, dark-green glyphs) and unbound NMDARs (larger, dark-blue glyphs). Snapshots **(A–C)** were made from data generated by MCell during a single trial and taken at 1, 200 μs, and 17 ms after glutamate release, respectively. Glutamate diffused rapidly throughout the synaptic cleft, binding to AMPARs and NMDARs. Singly bound AMPARs are shown as medium-green glyphs, doubly bound AMPARs as bright-green glyphs, open AMPARs as yellow glyphs, and desensitized AMPARs as black glyphs. Singly bound NMDARs are shown as medium-blue glyphs, doubly bound NMDARs as bright-blue glyphs, and open NMDARs as white glyphs. During this trial the peak of AMPAR activation occurred at ~200 μs and the peak of NMDAR activation occurred at ~17 ms when a single NMDAR flickered open (white glyph indicated by white arrow in **C**). **(D–F)** Time-lapse sequence of glutamate decay and calcium influx after stimulation. Snapshots for **(D–F)** were made from data generated by MCell during a single trial and taken at 100 μs, 17, and 100 ms after glutamate release, respectively. The presynaptic axon (green) on the right makes an en passant synapse with the postsynaptic spine (translucent yellow) on the left. Free glutamate molecules (cyan dots) diffuse from the synaptic cleft within a few microseconds and are taken up within a 100 ms by astroglial transporters (not visualized but present in the simulation). Some glutamate remains bound and activates NMDARs for many tens of microseconds allowing influx of calcium (white dots) as NMDARs flicker open and closed. In this example, calcium entered the spine through a single open NMDAR and was cleared from the spine head via calcium pumps in the spine membrane and by diffusion through the spine neck.

A visualization of simulated release and Ca^2+^ influx at spine #37 after an EPSP+bAP stimulus in the absence of calbindin is shown in Figure 8. Glutamate is released, diffuses, and activates receptors (Figures 8A–D). Ca^2+^ flows in through an opened NMDA receptor (Figure 8E) and is pumped out as the voltage decreases (Figure 8F). The visualization illustrates the level of detail that the simulations can reveal.

### Fate of calcium entering spines during a bAP and an EPSP+bAP stimulus

**Figure 9:**
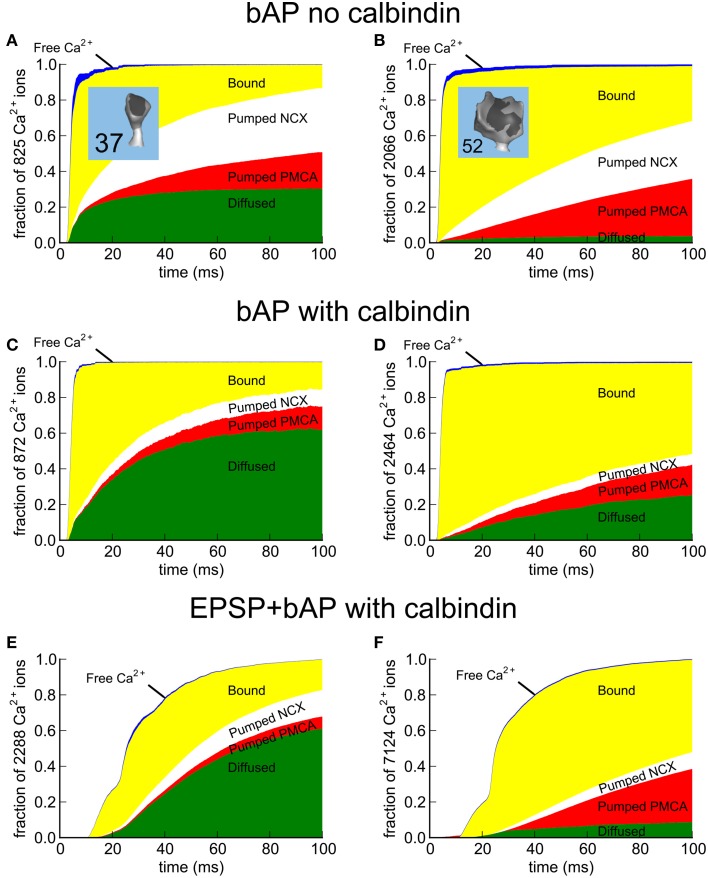
**Fate maps of Ca^2+^ entering spines #37 and #52 in response to EPSP+bAP**. The fate of all Ca^2+^ entering the spines was tracked for 100 ms after the stimulus. While inside the spine, Ca^2+^ can diffuse freely (blue) or bind to Ca^2+^-binding proteins and pumps (yellow). To restore the resting state, Ca^2+^ that enters is removed from the spine by PMCA pumps (red), NCX exchangers (white), or via diffusion through the spine neck (green) as free Ca^2+^ or while bound to mobile calbindin. **(A,C,E)** Fate of Ca^2+^ in median-size spine #37; **(A)** after a bAP in the absence of calbindin, **(C)** a bAP in the presence of calbindin, or **(E)** an EPSP+bAP in the presence of calbindin. **(B,D,F)** Fate of Ca^2+^ in large spine #52; **(B)** after a bAP in the absence of calbindin, **(D)** a bAP in the presence of calbindin, or **(F)** an EPSP+bAP in the presence of calbindin. Data is represented as a fraction of the total Ca^2+^ influx. The actual number of Ca^2+^ ions tracked is indicated in the y-axis label.

During the first 100 ms of a bAP stimulus, we monitored the fate of all the Ca^2+^ that entered the spine in the absence or presence of calbindin (Figures 9A–D). For comparison, we also monitored the fate of Ca^2+^ after an EPSP+bAP stimulus in the presence of calbindin (Figures 9E,F). The Ca^2+^ fates following the three stimuli are shown for a median-sized spine (#37, Figures 9A,C,E) and a large spine (#52; Figures 9B,D,F). Ca^2+^ was tracked after its influx through both VDCCs and NMDARs. We recorded its binding to CBPs, NCXs and PMCAs, and its eventual efflux out of the spine via the pumps and via diffusion through the neck into the shaft. The pumps and CBPs bound Ca^2+^ as soon as it entered the spine (yellow). Only a tiny fraction of Ca^2+^ remained free in the spine (blue). Ca^2+^ that bound to pumps was eventually pumped out of the spine (red and white) unless it unbound before being pumped. Ca^2+^ bound to immobile CBPs was released gradually to be captured by pumps or to diffuse out of the spine neck either while free or while bound to calbindin (green).

As expected, a larger fraction of total Ca^2+^ exits the median-size spine than the large spine by diffusion into the shaft. The principal effect of calbindin is to decrease the fraction of Ca^2+^ exiting via the pumps and to accelerate diffusion of Ca^2+^ into the shaft while it is bound to calbindin. Sabatini et al. (2002) reported that the length of time required for equilibration of Ca^2+^ between the spine and dendritic shaft during a bAP averaged 89 ± 31 ms, varying from 12 to 190 ms. They concluded that, in the absence of indicator (which diffuses faster than the endogenous CBPs), the time required for complete equilibration between the spine and shaft is likely to be longer than 1 s. Because the Ca^2+^ transient decays much faster than Ca^2+^ is cleared by diffusion, they concluded that diffusion through the spine neck is not likely to be a major determinant of the decay time of a Ca^2+^ transient produced by a bAP. Our simulations of Ca^2+^ movements are consistent with this conclusion.

## Discussion

One goal of this study was to perform a “computational” reconstitution assay. We tested whether the locations, estimated numbers, and kinetic behavior, determined *in vitro*, of the major proteins believed to control the influx and efflux of Ca^2+^ in spines can account for the measured size and decay time of Ca^2+^ transients in a computer reconstruction of intact dendrites and spines. We have shown that the properties of these proteins can indeed account for the size and decay of the Ca^2+^ transients when incorporated into our model. This conclusion comes with several qualifications. Some of the required parameters, in particular, the densities of individual classes of proteins in subcellular membranes, are difficult to measure experimentally. Hence, we have sometimes used molecular numbers, and/or membrane protein densities measured in non-neuronal cells, to establish biologically reasonable densities. For example, the densities of Na^+^/Ca^2+^ exchangers (NCXs) and plasma membrane Ca^2+^ ATPases (PMCAs) on the membrane of the spine head have not been measured directly. In our calibrations, we found that the required density of PMCAs in the spine head is higher by a factor of 2–3 than their measured density in plasma membranes of other cell types (see below). The values of the densities of NCXs and PMCAs that fit our calibration depend, in part, on their assigned turnover numbers. However, it is not possible to be certain that the enzymatic turnover numbers measured *in vitro* for these two pumps accurately reflect the turnover numbers *in vivo*. For example, if the turnover rate for PMCAs is higher *in vivo* than the rate measured *in vitro*, a lower density of PMCAs would be sufficient to account for the transients observed *in vivo*. Nonetheless, all of the final parameters in this model are within reasonable physiological limits. Thus, we conclude that the set of proteins we have used in the model, to a first approximation, can serve as the principal regulators of Ca^2+^ transients in spines. In addition, we conclude that the properties of these proteins, measured *in vitro* in the studies we have chosen (see Methods Section), are not likely to be grossly different than their properties displayed *in vivo*. Another area of experimental uncertainty is the immobile endogenous buffer, which represents the aggregate concentration and binding rates of the set of cytosolic proteins that bind Ca^2+^. The concentration (79.7 μM) and *K*_*D*_ of the CBP that we established during the calibration are reasonable considering the high total protein concentration in the cytosol, and the weak binding properties of most immobile Ca^2+^ binding proteins. Because the concentration of the immobile buffer is not well-constrained by experiments, one might wonder whether a different concentration of CBP's would result in an equally good fit with a different set of values for the other parameters. However, there was not another good fit within the range of each of the parameters that we searched (Table 2). Because the range of parameters that we searched was determined by the range of their values measured previously *in vitro*, we conclude that there are not multiple sets of physiologically realistic parameters that are compatible with a global fit of the cellular experimental data we have used.

The distribution of PMCA and NCX pumps between spine and shaft is qualitatively consistent with studies showing that Ca^2+^ pumps are distributed unevenly in dendrites and spines. In some situations, PMCA pumps appear to cluster at the neck and base of the spine head or in the PSD (Burette and Weinberg, 2007; Burette et al., 2010; Weinberg, personal communication). There have been no direct experimental estimates of the density of PMCA's on spine or dendritic membranes. However, in non-neuronal cells, PMCAs have been estimated to be 0.1% of plasma membrane proteins and 0.3–1% in neuronal cells (Stauffer et al., 1995). Quinn et al. (1984) estimated the density of total proteins in the plasma membrane of a BHK cell at 30,000 μm^−2^. Those numbers suggest that the neuronal membrane density of PMCA pumps would be estimated to be as high as 300 μm^−2^. Our final calibrated density of 998 μm^−2^ in the spine membrane is about three times that value. Biochemical estimates of densities of the pump on plasma membrane have some uncertainty because it is difficult to know what proportion of an isolated membrane fraction is plasma membrane, and because the measures by immunoblot of labeled phosphorylation intermediates are often semi-quantitative (Stauffer et al., 1995). Nonetheless, because of the importance of rapid Ca^2+^ transients in spines, one might expect them to have a higher PMCA density than BHK cells, particularly at the postsynaptic interface. A prediction of our model is that one of three things is true (or some combination): 1. PMCAs are present at a higher density in spines than in non-neuronal cells; 2. they have a higher turnover number *in vivo* than measured *in vitro*; or 3. there are additional factors important for removing Ca^2+^ from the spine that are not well-understood by molecular neurobiologists. We favor the idea that PMCAs are expressed at unusually high levels in the spine membrane because spines have evolved to exert tight control over Ca^2+^ fluxes.

The model presented here doesn't include several additional spine membrane components that can modulate the timing and/or amplitude of Ca^2+^ transients under particular conditions, or in particular synaptic types. These components include SK channels, calcium-permeable AMPARs, metabotropic receptors, and inositol trisphosphate (IP3) and ryanodine receptors in SER. SK channels hyperpolarize the spine membrane in response to an initial Ca^2+^ influx, and thus strongly modulate the influx of Ca^2+^ during repetitive stimulation (Ngo-Anh et al., 2005). They do not have a large effect during a single bAP or EPSP+bAP. Their influence on Ca^2+^ influx during the kinds of repetitive stimuli that lead to synaptic plasticity will be important to add to this model for future studies. Ca^2+^-permeable AMPARs are believed to play a role in excitatory synapses onto inhibitory GABAergic neurons (Mahanty and Sah, 1998; Laezza and Dingledine, 2004). Under some circumstances, they may also play a transient role in Ca^2+^ influx into spine synapses during consolidation of LTP (Plant et al., 2006). They don't appear to be an important source of Ca^2+^ influx in the majority of glutamatergic synapses onto excitatory neurons. Ca^2+^ stores in SER containing IP3 or ryanodine receptors are present in a small proportion of spine synapses in the hippocampus (~20%). We have not modeled those spines in the present study. It will be interesting to examine the effect of the presence of Ca^2+^-permeable AMPARs and Ca^2+^ stores on spine Ca^2+^ transients in future studies.

Our model of Ca^2+^-handling permits accurate simulation of diffusion of Ca^2+^ and mobile Ca^2+^-binding proteins through spine necks and into the nearby dendritic shafts, assuming a fairly uniform cytosol. However, we have not included agents that might mediate changes in the diffusional barrier in the spine neck, such as highly cross-linked actin cytoskeleton or mobile organelles that might move transiently into the neck (Bloodgood and Sabatini, 2005). Thus, our results do not reflect these influences.

We found that the single exponential decay of the fluorescence of Fluo-4 and OGB1 observed in the experiments of Sabatini et al. (2002) constrained the values of the on-rate and the *K*_*D*_ of the immobile CBP. When the on-rate of the immobile CBP was set significantly slower than the on-rates of Fluo4 or OGB1, the decay of the simulated fluorescence transient (i.e., Ca^2+^-bound to the indicator) was best fit by two exponential curves, one fast and one slow. As the on-rate of the immobile CBP was increased and approached the on-rate of the indicator, the shape of the decay phase of the fluorescence transient was better fit by a single exponential. Furthermore, the off-rate (and therefore the overall *K*_*D*_) of the immobile CBP also had to be balanced against that of the indicator to avoid a biphasic exponential decay of the fluorescence transient. This phenomenon can be understood by noting that during the rising phase of a transient, the buffer or dye with the higher on-rate will initially bind most of the Ca^2+^, but during the decay phase the buffer or dye with the lower *K*_*D*_ (i.e., highest affinity and slowest off-rate) will eventually bind the most Ca^2+^ (Markram et al., 1998). The best-fit parameters we found for the immobile CBP allows the indicator to bind and retain most of the Ca^2+^ throughout the fluorescence transient while also fitting the experimental measurements of endogenous buffering capacity.

To understand and model the biochemical responses to Ca^2+^ in the postsynaptic spine, including the interaction of Ca^2+^ with calmodulin and its target proteins, it is important to know the precise height and time course of the Ca^2+^ transients. Since our simulations allow us to measure free [Ca^2+^], with or without added indicator and with or without lowpass filtering, we were able to measure both the perturbation of free Ca^2+^ due to the added indicator, and the error in the estimation of free Ca^2+^ based on the fluorescence signal (Figures 6E,F). The error in the estimation arises from two factors: (1) the estimation method requires the assumption that free and indicator-bound Ca^2+^ are in quasi-equilibrium; in other words, the indicator binds Ca^2+^ fast enough to track the Ca^2+^ transient accurately; and (2) the estimation method assumes that the data acquisition and subsequent lowpass filtering of the fluorescence signal are fast enough to track the fluorescence transient. The model shows that both of these assumptions are false. Ca^2+^ does not approach equilibrium with the indicator during the transient, and the Ca^2+^ peak is significantly attenuated by the lowpass filter.

The comparison of Ca^2+^ fluxes into a median size spine and a large spine (Figures 6E,F, **9**) reveal that, although a larger amount of Ca^2+^ flows into the larger spine, the effective concentration of free Ca^2+^ reached in that spine is smaller because the Ca^2+^ is averaged over a much larger volume. Our future studies will examine in more detail, in different sizes and shapes of spines, the relationship between the surface to volume ratio of a spine and the concentrations and local interactions of Ca^2+^ in the spine cytosol after a stimulus.

The model presented here will be a useful adjunct to experimental studies of the regulatory systems that control synaptic strength. For example, the basic model of Ca^2+^ handling in spines provides a test bed in which the sensitivity of Ca^2+^ transients to changes in the numbers and parameters of other modulating proteins can be predicted both independently and in combination. The model will also enable future studies in which calmodulin and its target enzymes will be added to the spine cytosol to test and predict how Ca^2+^ influx, triggered by a wide variety of stimuli, can affect activation of Ca^2+^-regulated enzymes that control synaptic plasticity.

### Conflict of interest statement

The authors declare that the research was conducted in the absence of any commercial or financial relationships that could be construed as a potential conflict of interest.
